# General low-temperature growth of two-dimensional nanosheets from layered and nonlayered materials

**DOI:** 10.1038/s41467-023-35983-6

**Published:** 2023-01-19

**Authors:** Biao Qin, Muhammad Zeeshan Saeed, Qiuqiu Li, Manli Zhu, Ya Feng, Ziqi Zhou, Jingzhi Fang, Mongur Hossain, Zucheng Zhang, Yucheng Zhou, Ying Huangfu, Rong Song, Jingmei Tang, Bailing Li, Jialing Liu, Di Wang, Kun He, Hongmei Zhang, Ruixia Wu, Bei Zhao, Jia Li, Lei Liao, Zhongming Wei, Bo Li, Xiangfeng Duan, Xidong Duan

**Affiliations:** 1 Hunan Provincial Key Laboratory of Two-Dimensional Materials, State Key Laboratory for Chemo/Biosensing and Chemometrics, Advanced Semiconductor Technology and Application Engineering Research Center of Ministry of Education of China, Changsha Semiconductor Technology and Application Innovation Research Institute, College of Semiconductors (College of Integrated Circuits), School of Physics and Electronics, Hunan University, Changsha, 410082 China; 2 State Key Laboratory for Chemo/Biosensing and Chemometrics, College of Chemistry and Chemical Engineering, Hunan University, Changsha, 410082 China; 3 State Key Laboratory of Superlattices and Microstructures, Institute of Semiconductors, Chinese Academy of Sciences, Beijing, 100083 China; 4 Shenzhen Research Institute of Hunan University, Shenzhen, 518063 China; 5 Department of Chemistry and Biochemistry, University of California, Los Angeles, CA USA

**Keywords:** Two-dimensional materials, Synthetic chemistry methodology, Electronic and spintronic devices

## Abstract

Most of the current methods for the synthesis of two-dimensional materials (2DMs) require temperatures not compatible with traditional back-end-of-line (BEOL) processes in semiconductor industry (450 °C). Here, we report a general BiOCl-assisted chemical vapor deposition (CVD) approach for the low-temperature synthesis of 27 ultrathin 2DMs. In particular, by mixing BiOCl with selected metal powders to produce volatile intermediates, we show that ultrathin 2DMs can be produced at 280–500 °C, which are ~200–300 °C lower than the temperatures required for salt-assisted CVD processes. In-depth characterizations and theoretical calculations reveal the low-temperature processes promoting 2D growth and the oxygen-inhibited synthetic mechanism ensuring the formation of ultrathin nonlayered 2DMs. We demonstrate that the resulting 2DMs exhibit electrical, magnetic and optoelectronic properties comparable to those of 2DMs grown at much higher temperatures. The general low-temperature preparation of ultrathin 2DMs defines a rich material platform for exploring exotic physics and facile BEOL integration in semiconductor industry.

## Abstract (web summary)

Chemical vapor deposition (CVD) is a versatile method to synthesize 2D materials, but usually requires high growth temperatures. Here, the authors report a BiOCl-assisted CVD approach to grow 2D nanosheets from 27 different layered and nonlayered materials at temperatures <500 °C, which are compatible with back-end-of-the-line industrial processes.

## Introduction

Two-dimensional materials (2DMs) with atomic thickness, including 2D layered materials (LMs) and nonlayered materials (NLMs), have attracted intense recent interest for their unique and rich physical and chemical properties, such as ultrathin geometry, high surface atom ratio, semiconductivity, superconductivity, magnetic properties, charge density waves and significant potential for applications in electronics, sensors, and energy storage devices^1–5^. The 2DMs explored to date are largely derived from intrinsically LMs. Although there have been occasional reports of 2DMs from NLMs, the general synthesis of ultrathin 2DMs, particularly from NLMs, has been challenging. NLMs whose number far exceeds that of LMs exhibit abundant physical and chemical properties^6^. Therefore, the synthesis of rarely reported 2DMs, especially 2D NLMs, is of great significance for basic research and practical technologies.

Chemical vapor deposition (CVD) methods, such as the molten salt-assisted method^7^, space-confined method^8^, and reverse flow method^9,10^, have been extensively utilized in synthesizing various ultrathin 2DMs and corresponding heterostructures. However, most of these synthetic approaches are highly specific to a given class of materials and often require excessively high temperature (e.g., >700 °C), which is not compatible with the traditional front-end of line transistor (FEOL) (700 °C) or back-end-of-line (BEOL) (450 °C) fabrication process in semiconductor industry^11^. Though low-temperature growth of 2DMs is possible using metal-organic precursors with high volatility^12–14^, the relevant growth requires high airtightness of the equipment, and generally speaking, the product is highly toxic. The growth of 2DMs using metal or metal oxide precursors is low-cost, environmentally friendly and extensively investigated. High melting/volatile point of the metal precursors in CVD methods represents one of the key challenges for low-temperature growth of 2DMs. To this end, Zhou et al. successfully developed a molten salt-assisted CVD method and found that metal oxides can react with salt to form metal oxychlorides with high evaporation concentrations, which is favorable for decreasing the synthetic temperature and achieving ultrathin 2DMs^15^. However, the synthesis temperature in most of these methods remains above 600 °C and in many cases exceeding 700 °C. Thus, it is highly desirable to develop general methods amenable for the synthesis of ultrathin 2DMs using metal or metal oxide precursors at a considerably lower growth temperature (e.g., <700 °C for FEOL and <450 °C for BEOL), which is essential for facile integration of with conventional semiconductor industry.

Herein, we report a BiOCl-assisted CVD method for general synthesis of ultrathin 2DMs at a low growth temperature largely compatible with BEOL process. BiOCl is an environmentally friendly and low-cost basic salt^16,17^, and we found that it can effectively reduce the volatilization temperature of diverse metal precursors and enable the growth of most 2DMs from both LMs and NLMs at a low temperature range 280–500 °C. Our systematic studies demonstrate the low-temperature process is beneficial to 2D growth of ultrathin 2DMs and the preferential adsorption of oxygen on the surface of NLMs suppresses isotropic growth promote the formation of a 2D morphology from NLMs. Our study defines a general BiOCl-assisted CVD method for synthesis of a series of 2DMs at a relatively low-temperature, thus providing a rich 2DMs platform for fundamental studies and facile integration with traditional semiconductor technologies.

## Results

### General low-temperature growth of 2D nanosheets

**Fig. 1:**
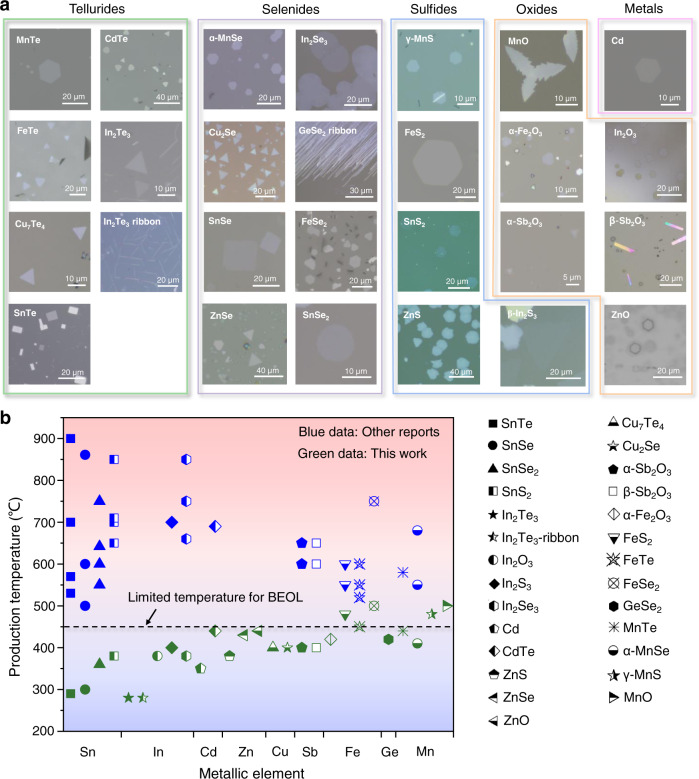
A library of 2D materials produced at low temperature (substrate and metal source temperature). **a** Optical microscopy images of the 27 synthesized ultrathin 2D tellurides, selenides, sulfides, oxides and metals. Contains: telluride (MnTe, FeTe, Cu_7_Te_4_, SnTe, CdTe, In_2_Te_3_, In_2_Te_3_ ribbon), selenide (MnSe, FeSe_2_, Cu_2_Se, SnSe, SnSe_2_, GeSe_2_ ribbon, ZnSe, In_2_Se_3_), sulfide (*γ*-MnS, FeS_2_, SnS_2_, ZnS, *β*-In_2_S_3_), oxide (MnO, *α*-Fe_2_O_3_, *α*-Sb_2_O_3_, *β*-Sb_2_O_3_, ZnO, In_2_O_3_), metal (Cd). **b** Comparison of synthesis temperature of the material library with other reports. Limited temperature is 450 °C for BEOL. The blue data is other reports, green data is this work. Detailed references are shown in Supplementary Table 1.

In a typical BiOCl-assisted growth, selected metals are mixed and grounded with BiOCl powder in a proper ratio to form a uniform mixture as precursors for the CVD growth of the selected 2DMs at low temperature (Methods, Supplementary Figs. 1 and 2). Using this method, we synthesized a series of ultrathin 2DMs on mica substrates, including a diverse family of materials including tellurides, selenides, sulfides, and oxides to metal, with a total of 27 2D LMs and NLMs crystals from groups VIIB (Mn), VIII (Fe), IB (Cu), IIB (Zn, Cd), IIIA (In), IVA (Sn, Ge) and VA (Sb). The optical microscopy (OM) images of the products demonstrated various morphologies of triangles, hexagons, rectangles, stars, and ribbons, respectively (Fig. 1a). And we achieved the preparation of 1 × 1 cm^2^ continuous uniform 2D In_2_S_3_ film on mica substrate (Supplementary Fig. 3). Among them, MnTe, FeTe, *α*-Fe_2_O_3_, Cu_7_Te_4_, SnTe, CdTe, MnSe, Cu_2_Se, FeSe_2_, ZnSe, FeS_2_, *γ*-MnS, β-In_2_S_3_, ZnS, *α*-Sb_2_O_3_, *β*-Sb_2_O_3_, ZnO, MnO and In_2_O_3_ are 2D NLMs, and the others are LMs (Supplementary Figs. 4–8). Moreover, to the best of our knowledge, ultrathin Cu_7_Te_4_, In_2_Te_3_, In_2_Te_3_ ribbon, ZnSe, *γ*-MnS, GeSe_2_ ribbon, ZnS, Cu_2_Se, MnO, *α*-Fe_2_O_3_, ZnO, In_2_O_3_ and Cd have been rarely reported before.

Notably, most 2DMs were synthesized at a low temperature below 450 °C (limited temperature for BEOL), which cannot be achieved with traditional approaches (Fig. 1b). In general, the synthesis temperature (substrate and metal source temperature) using the BiOCl-assisted method is approximately 200–300 °C lower than that of the traditional CVD process (Supplementary Table 1), demonstrating BiOCl-assisted method has lower energy consumption, lower equipment requirements, more compatible with traditional CMOS process and can minimize potential thermal damage of the ultrathin nanosheet product in a possible multistep thermal treatment process^9^.

The thickness of the synthesized nanosheets was characterized by atomic force microscopy (AFM), and the results showed that most of the thinnest nanosheets were less than 10 nm, and some were even monolayer and bilayer (Supplementary Fig. 9). Compared with the reported thinnest samples grown by traditional CVD (Supplementary Fig. 10, Supplementary Table 2), most of the samples synthesized by the BiOCl-assisted method are thinner, indicating that our method has significant advantages in preparing ultrathin 2DMs. Raman spectra are used to probe the lattice vibration of these 2DMs (Supplementary Figs. 11–13), and each prominent Raman peak coincides perfectly with previous reports.

### Controlled growth of 2D nanosheets

**Fig. 2:**
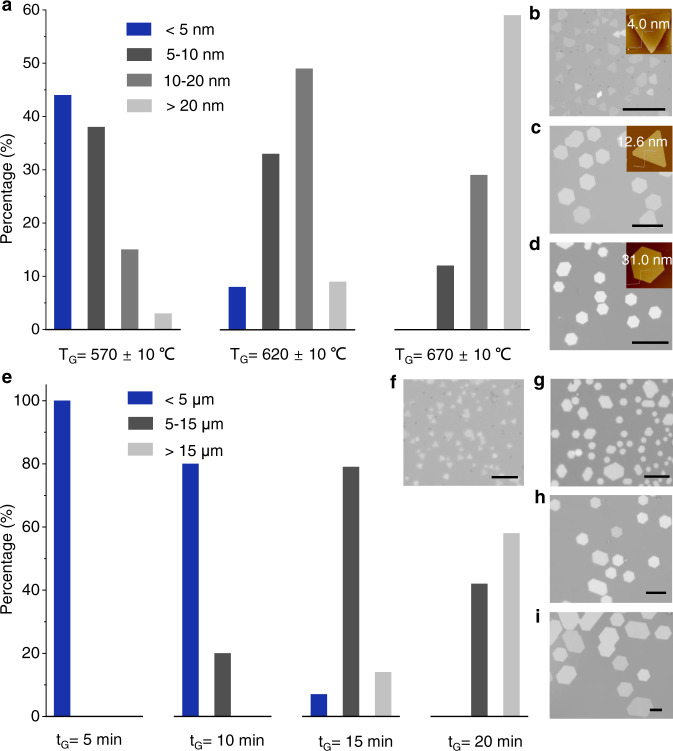
Thickness-tunable and size-tunable synthesis of nanoplates by varying the growth temperature (T_G_) and growth time (t_G_). **a** The statistical thickness distributions of the MnS nanosheets synthesized with T_G_ of 570 ± 10 °C, 620 ± 10 °C, and 670 ± 10 °C, respectively. **b**–**d** Typical OM images and AFM images (inset) of the MnS nanosheets synthesized with T_G_ of 575 °C (**b**), 630 °C (**c**), and 680 °C (**d**), respectively, under a constant carrier gas flow at 140 sccm (Ar/H_2_ mixture with 2% H_2_). Scale bar: 20 μm. **e** The statistical size distributions of the MnS nanosheets synthesized with t_G_ of 5 min, 10 min, 15 min and 20 min at 600 °C, respectively. **f**–**i** Typical OM images of the MnS nanosheets synthesized with t_G_ of 6 min (**f**), 10 min (**g**), 15 min (**h**), and 20 min (**i**), respectively. Scale bar: 10 μm.

The physical and chemical property of 2DMs depends on their thickness and size. It is essential to precisely control the crystal size and thickness of 2DMs during the growth process. As a demonstration, we focus on the controllable synthesis of MnS and *α*-Fe_2_O_3_ nanosheets. By varying the growth temperature (T_G_) while keeping other conditions (e.g., flow rate) constant, we found that the thickness of the produced nanosheets (Fig. 2b–d) show a systematic evolution with the increase of T_G_. In particular, the average thicknesses of the MnS nanosheets are controlled to be 0–10, 5–20 and above 20 nm when the T_G_ is 570 ± 10, 620 ± 10 and 670 ± 10 °C, respectively (Fig. 2a). The thickness of *α*-Fe_2_O_3_ nanosheets can also be tuned by T_G_ in a similar manner (Supplementary Fig. 14). Additionally, the size of MnS nanosheets gradually increased with the increasing of is largely dictated by the edge energetics (Fig. 2e–i). The rapid attachment of the precursor atoms to the energetic growth edge of the 2D nanosheets expands the 2D crystal laterally. On the other hand, when the T_G_ is higher, the growth is more thermodynamically controlled and thicker nanosheets are more likely to be produced^18^. In this regard, the ability to conduct low temperature growth is also beneficial for preparing ultrathin 2DMs.

### Structural characterizations of 2D nanosheets

**Fig. 3:**
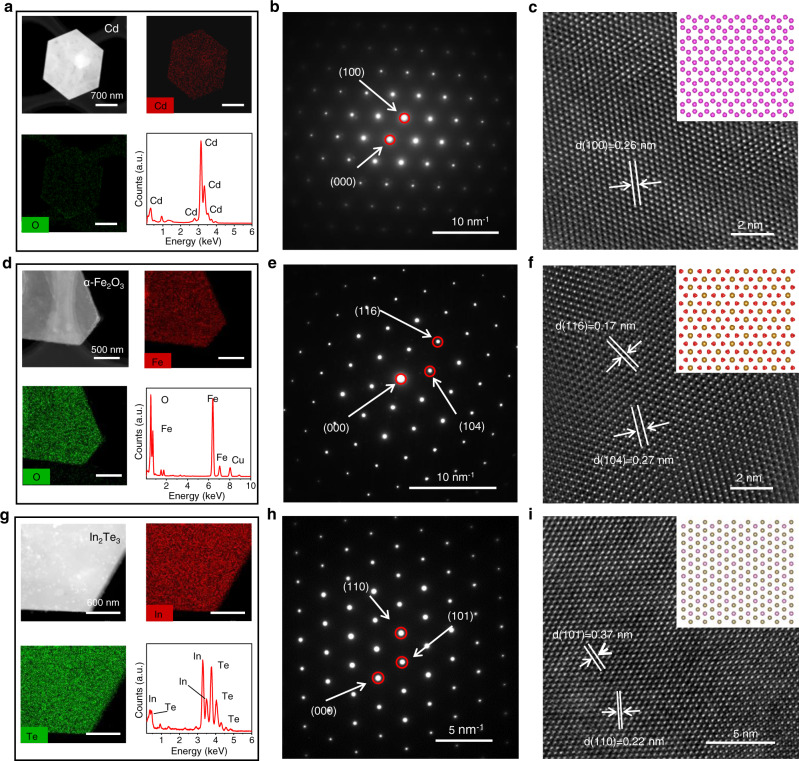
Crystal structure of 2D metals, oxides, and tellurides. **a** HAADF-TEM image, TEM-EDS mapping of Cd and O, and EDS of Cd nanosheets, respectively. **b** SAED pattern of Cd nanosheet. **c** HRTEM image of Cd nanosheet. The inset shows the corresponding atomic models. **d**–**f** Image of *α*-Fe_2_O_3_ nanosheet HAADF-TEM, TEM-EDS mapping, EDS, SAED pattern, HRTEM (inset is the corresponding atomic model), respectively. **g**–**i** Image of In_2_Te_3_ nanosheet HAADF-TEM, TEM-EDS mapping, EDS, SAED pattern, HRTEM (inset is the corresponding atomic model).

High-resolution transmission electron microscopy (HRTEM) was used to investigate the crystal structure of the nanosheets. High-angle annular dark-field scanning transmission electron microscopy (HAADF-TEM) images of Cd nanosheets transferred on the copper grid (Fig. 3a) show a regular hexagon, and the corresponding transmission electron microscopy energy-dispersive spectroscopy (TEM-EDS) mapping indicates that Cd is uniformly distributed with trace oxygen. The corresponding EDS spectrum shows the characteristic peaks of Cd atoms. The selected area electron diffraction (SAED) pattern (Fig. 3b) and the corresponding HRTEM image (Fig. 3c) reveal that the nanosheet has a lattice spacing of 0.26 nm assigned to the (100) plane, which is consistent with the crystal structure of Cd (inset in Fig. 3c). Similarly, we obtained HAADF-TEM images of 2D oxide Fe_2_O_3_ and telluride In_2_Te_3_ and their corresponding TEM-EDS mappings (Fig. 3d, g). The corresponding EDS spectra show that both the Fe:O and In:Te atomic ratios are 2:3. The SAED pattern (Fig. 3e, h) and HRTEM images (Fig. 3f, i) of Fe_2_O_3_ and In_2_Te_3_ nanosheets show a hexagonal arrangement. The lattice spacings of Fe_2_O_3_ (Fig. 3f) are 0.17 nm and 0.27 nm, denoting the (116) and (104) planes of the hexagonal structure, respectively. The lattice spacings of In_2_Te_3_ (Fig. 3i) are 0.22 nm and 0.37 nm, referring to the (110) and (101) planes of the hexagonal structure, respectively. These results are also in accord with the corresponding crystal structures (inset in Fig. 3f, i). The detailed HAADF-TEM, TEM-EDS mapping, EDS spectrum, HRTEM, and SAED of other 2DMs are shown in Supplementary Figs. 15–26.

### Mechanism for BiOCl-assisted low temperature growth

**Fig. 4:**
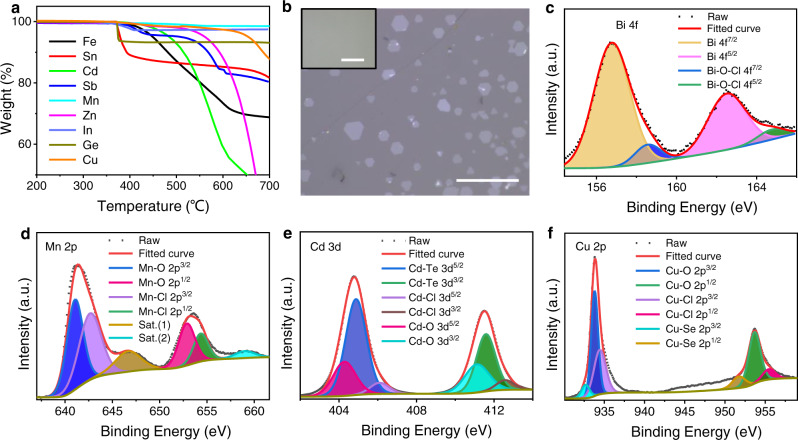
Low temperature growth mechanism of the BiOCl-assisted CVD method. **a** Thermogravimetric analysis after mixing metal and basic salt BiOCl homogeneously. **b** Comparison of the growth of MnSe with and without BiOCl (inset) under the same growth conditions. Scale bar: 40 μm. **c**–**f** XPS of the transition state in the process of salt-assisted growth of 2D materials. XPS spectra of Bi 4 f, Mn 2p, Cd 3d, and Cu 2p, shown in **c**–**f**, respectively.

We further performed thermogravimetric analysis, X-ray photoelectron spectroscopy (XPS) and EDS to investigate the growth mechanism of the BiOCl-assisted CVD method. The vaporization (weight loss) temperature and process of the thoroughly ground and mixed metal precursors with BiOCl was characterized by thermogravimetric analysis. The results showed that the vaporization temperature of most of the mixed precursor is between 350 °C and 500 °C, which is also consistent with the growth temperature of the synthesized 2DMs (Fig. 4a). It is proved that the volatilization temperature of metal oxychloride temperature in the assistance of BiOCl is lower than that of the assistance of NaCl^15^.

Subsequently, as an example, the growth of MnSe through CVD with and without BiOCl assistance (Fig. 4b) showed that regular-shaped MnSe nanosheets can be realized in BiOCl-assisted growth, while nothing is deposited in the process without BiOCl (inset in Fig. 4b), indicating that BiOCl can efficiently promote the low-temperature growth of 2DMs. We also used *α*-Fe_2_O_3_ growth to verify the significance of BiOCl in low-temperature growth (Supplementary Fig. 27). It is noteworthy that we successfully synthesized ultrathin SnTe nanoplates on SiO_2_/Si substrates with the assistance of BiOCl (Supplementary Fig. 28), even though it was difficult to achieve in previous reports^19^. The growth of 2D materials on temperature-sensitive substrates is of great significance, and we successfully fabricated 2D SnSe_2_ nanosheets on polyimide (PI) substrates (Supplementary Fig. 29). These comparative studies and analyses confirmed the high efficiency of BiOCl-assisted method in the low-temperature growth of 2DMs.

The composition and chemical states of the intermediate during the growth process were further analyzed using XPS. Here, after short-term growth, the system was rapidly cooled, and the intermediate was obtained (details in Methods). XPS data of the intermediate showed the Bi *4* *f* are mainly Bi *4* *f*^7/2^ and Bi *4f*^5/2^, followed by the smaller Bi-O-Cl *4f*^7/2^ and Bi-O-Cl *4f*^5/2^ bond signals (Fig. 4c), suggesting the Bi is primarily in the form of elemental substance (Bi(0)) with a small amount of BiOCl state^20,21^. The elemental Mn peaks in the XPS data of the intermediate products of the MnSe nanosheet growth process demonstrated the presence of Mn-O and Mn-Cl bond signals (Fig. 4d)^22–24^. The result demonstrated the existence of transition state MnO_a_Cl_b_. It should be pointed out that sat. (1) and sat. (2) are satellite lines with two component peaks (Fig. 4d)^25^. As more examples, in the XPS data of the intermediate products of the CdTe nanosheet growth process, the Cd-Te, Cd-Cl and Cd-O bond signals emerge in Cd *3d* peaks (Fig. 4e)^26–30^, confirming the existence of the transition state CdO_c_Te_d_Cl_e_. In the XPS data of the intermediate products of the Cu_2_Se nanosheet growth process show the presence of Cu-O, Cu-Cl and Cu-Se bond signals, confirming the emergence of transition state CuO_f_Se_g_Cl_h_ (Fig. 4f). Thus, we concluded that transition states, such as Bi, MO_i_Cl_j_ and MO_k_X_l_Cl_m_, generally exist in the growth process. Finally, we should note that there is no Bi element in the final nanosheets, which can be proven by SEM-EDS (Supplementary Figs. 30–35) and TEM-EDS (Supplementary Figs. 15–26). We found elemental Bi at the downstream end of the tube furnace outside the reaction zone (Supplementary Fig. 36).

According to the above discussion, we proposed a possible reaction mechanism for the growth of metals (M), oxides (MO), and chalcogenides (MX, X denote tellurium, selenium, and sulfur). The BiOCl can act as a promoter for the reaction. Metal precursors react with BiOCl to produce highly volatile MO_i_Cl_j_^15^. Thus, the element M can be transferred in the vapor state to the position of the substrate and react with H_2_ to produce M_k_O_l_ or M nanosheets. When chalcogen vapor (X) is supplied, MO_i_Cl_j_ reacts with chalcogen vapor to produce MO_k_X_l_Cl_m_ and can be transferred in the gas state to the position of the substrate. Then, with the help of H_2_, M_p_X_q_ nanosheets can be deposited on the substrate. Overall, with the aid of the BiOCl promoter, the metal precursor can be gasified, and the reaction can proceed at a relatively low temperature. Compared to the salt-assisted CVD process^15^, the volatilization temperature of precursors is 200–300 °C lower, which is essential for producing volatile intermediates to enable the growth of the selected 2DMs at lower temperature.

### Mechanism for the growth of ultrathin 2D NLMs

**Fig. 5:**
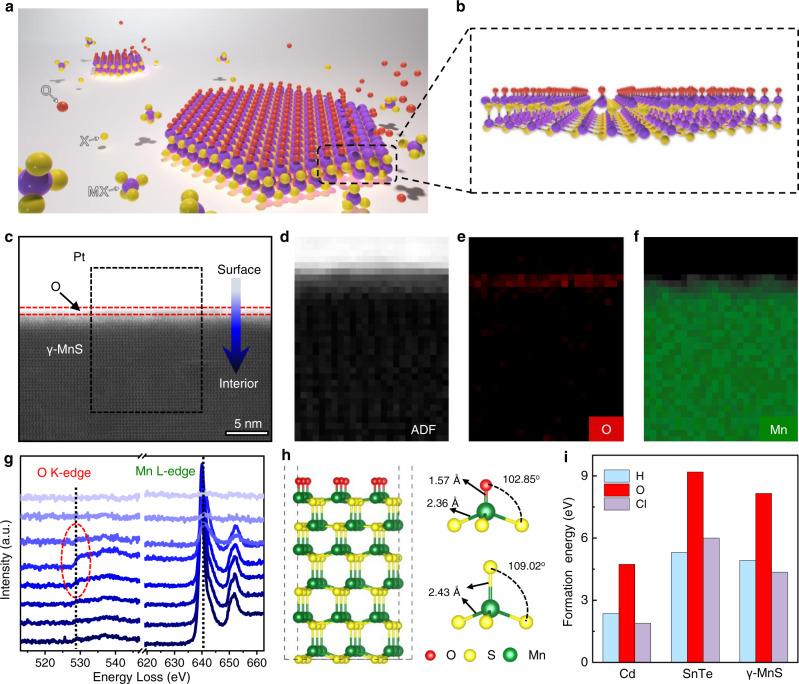
Oxygen-inhibited mechanism epitaxial growth of ultrathin 2D NLMs. **a** Schematic diagram of the growth mechanism. Purple, M (metal); yellow, X (sulfur group); red, O. **b** Front view of the material synthesized by the adsorption of oxygen atoms. **c** HAADF-STEM image of the cross-section of MnS nanosheets. **d**–**f** Annular dark field (ADF) image (**d**), the corresponding distribution of O element (**e**) and Mn element (**f**) using multiple linear least squares (MLLS) fitting. **g** EELS spectra corresponding to the O K-edge (left vertical position) and Mn L-edge (right vertical position) from the surface to the interior region. The red dotted frame is the surface positions of the nanosheets. **h** Schematic view of the distortion O-terminated MnS (001) surface. **i** Formation energy of adsorbing H, O and Cl on the surface of Cd, SnTe and MnS.

Different from LMs with weak vdW forces between layers, NLMs usually have a 3D crystal structure bonded with strong covalent forces, which makes it much more difficult to achieve anisotropic growth of 2D crystals. We find that our strategy is more facile to synthesize 2D NLMs via a unique oxygen-inhibited mechanism (Fig. 5a, b). To understand the growth mechanism of ultrathin 2D NLMs, scanning transmission electron microscopy (STEM) was used to characterize the synthesized 2D nonlayered MnS at the atomic scale (Fig. 5c). A typical annular dark field (ADF) image and the corresponding element mapping further revealed the spatial distribution of O elements on the surface (Fig. 5b–d). Electron energy loss spectroscopy (EELS) is used to investigate the spatial distribution of O and Mn elements near the surface by recording the O *K*-edge and Mn *L*-edge of MnS from the surface to the interior region (Fig. 5g)^31^. The intensity of the O *K*-edge peak (~528 eV) emerged on the surface (marked with a red dashed line in Fig. 5c) and disappeared inside the nanosheet (Fig. 5g). We further compare the STEM and EELS of 2D MnS grown by the common method and BiOCl-assisted growth method. After the samples are prepared by these two methods through CVD, Pt films are evaporated quickly, and then the samples are stored in an oxygen free and anhydrous environment until STEM cross-sections measurement are taken. The results show the presence of interfacial oxygen element in our method, but almost no oxygen element in the conventional method (Supplementary Fig. 37). These data directly confirm the oxygen-inhibited synthetic mechanism. In addition, we compared the thickness distribution of 2D MnS between the BiOCl-assisted growth method and the common method under the same conditions, and the results clearly showed that the oxygen-inhibited synthetic mechanism is highly efficient in preparing thin samples (Supplementary Fig. 38). Meanwhile, the HAADF-STEM atomic structure and the surface EELS analysis of MnSe nanosheets also confirm the existence of oxygen atoms on the surface (Supplementary Fig. 39). In addition, the SEM-EDS mapping (Supplementary Figs. 40 and 41) of the intermediate also revealed the existence of oxygen, consistent with the XPS analysis. the surface oxygen species the 2D NLMs is likely orientated from the promoter BiOCl.

Furthermore, we performed density of functional theory (DFT) calculations to confirm the absorption of the O atoms on the surface of 2D NLMs. Considering all possible gaseous elements, such as H, O and Cl, we calculated and compared the ground-state energies of the NLMs Cd, SnTe and MnS with surface adsorbed H, O and Cl atoms (Fig. 5h, Supplementary Fig. 42), and found the surface adsorption of O atoms is the most stable state for Cd, SnTe and MnS (Fig. 5i). The schematic view of the O-terminated MnS structure (Fig. 5h) shows that the adsorbed O atoms associated with the Mn atoms form a stable tetrahedral structure on the surface. These results indicate that O elements are more likely to bind with these NLMs on the surface, which is consistent with our experimental results.

Previous studies have reported that the adsorbed atoms on the surface of materials will mostly limit the continuous growth of materials^7,32–34^. During the growth process, the absorption of O on the surface can suppress the growth of nanosheets in the vertical direction, and atoms are added on the lateral edge of the nanosheet to promote the growth of nanosheets in the planar direction. In this regard, the existence of oxygen atoms on the surface of 2D NLMs is important for the highly anisotropic growth of these materials and finally the formation of nanosheets with 2D geometry.

### Physical property of the resulting 2DMs

**Fig. 6:**
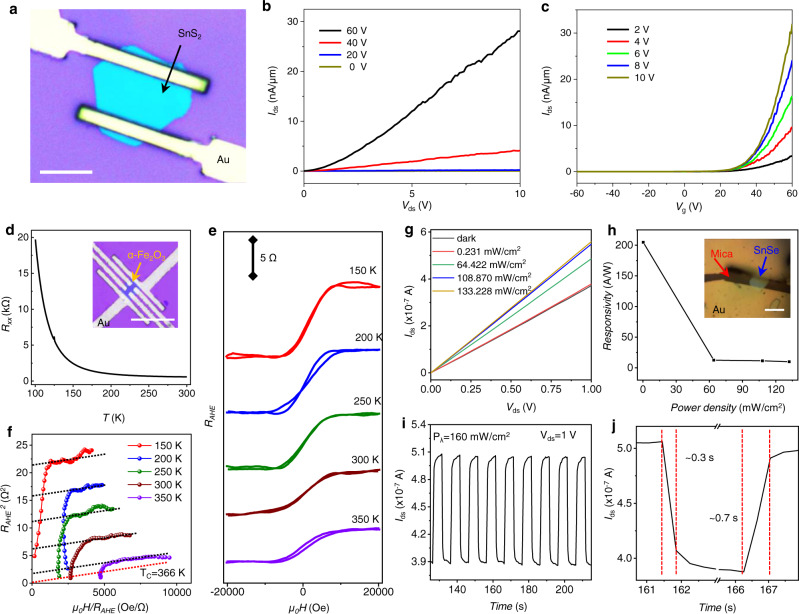
Electrical, Magnetic and optoelectronic characterizations of 2D nanosheet. **a** OM image of the SnS_2_ FET device, Scale bar: 10 μm. **b** Typical drain-source current (*I*_ds_) vs drain-source voltage (*V*_ds_) output characteristics of the SnS_2_ FET measured under various gate voltages. **c** Typical transfer curves of the FET. **d** The longitudinal resistance (*R*_XX_) at different temperatures for *α*-Fe_2_O_3_ nanosheet. The inset is OM image of the Hall bar device, Scale bar: 30 μm. **e** The resistance of anomalous Hall effect (*R*_AHE_) plotted as a function of the magnetic field at various temperature. **f** Arrott plots of the Hall resistance data of the *α*-Fe_2_O_3_ nanosheet shown in **e**. The dashed lines represent the intercept of *R*_AHE_^2^. **g** *I*_ds_–*V*_ds_ output characteristics of the SnSe photodetector in the dark and various laser powers with 808 nm laser. **h** The photodetector responsivity versus laser power densities with a voltage bias of 1 V. The inset is OM image of the photodetector, Scale bar: 10 μm. **i** Time dependent photoresponse of SnSe photodetector under 808 nm laser. Voltage bias is 1 V. **j** A typical time-dependent photoresponse curve in a quick scan mode. The vertical dotted lines represent the beginning and end moments of the photoresponse.

To evaluate the quality of the low-temperature grown 2D nanosheets, we fabricated various devices and evaluate their electronic and magnetic properties from the resulting materials. For example, the SnS_2_ nanosheet (synthesized at 450 °C) was used to construct a field effect transistor (FET) (Fig. 6a), that show an expected n-type transistor behavior (Fig. 6b) with a current ON/OFF switching ratio of 5 × 10^4^ (Fig. 6c). Overall, the device displayed a performance compares well with those synthesized at higher temperature reported previously^35,36^. In addition, we have investigated magnetotransport property of *α*-Fe_2_O_3_ nanosheets (synthesized at 520 °C) using a standard Hall bar device (insets in Fig. 6d). The longitudinal resistance (*R*_XX_) increased with the decreasing of temperature, demonstrating that the *α*-Fe_2_O_3_ nanosheet has the semiconducting feature (Fig. 6d). The Hall resistance, *R*_XY_, was measured under an external magnetic field, *μ*_*0*_*H*, applied perpendicular to the sample plane. For a magnetic material, R_XY_ can be decomposed into two parts:

$${R}_{{XY}}={R}_{{NHE}}+{R}_{{AHE}}$$

where *R*_NHE_ = *R*_0_*μ*_0_*H*, the resistance of normal Hall effect, and *R*_AHE_ = *R*_S_*M*_⊥_, the resistance of anomalous Hall effect; R_0_ and R_S_ are coefficients characterizing the strength of *R*_NHE_ and *R*_AHE_, respectively, and *M*_⊥_ is the sample magnetic moment perpendicular to the sample surface. The R_XY_ showed negligible or very small hysteresis from 150 K to 350 K (Fig. 6e). As the hysteresis is not clear, we used Arrott plot to confirm the FM behavior and to determine the Curie temperature of the *α*-Fe_2_O_3_ nanosheet (Fig. 6f). The positive (negative) values of the intercepts to the R_AHE_ axis indicate the ferromagnetic (paramagnetic) state. The linear intercept decreases with increasing temperature and approaches zero when the Curie temperature is reached^37^. In this way, a T_C_ of 366 K was determined for the *α*-Fe_2_O_3_ nanosheet when the intercept goes to zero (Fig. 6f). The result demonstrated that *α*-Fe_2_O_3_ nanosheet is a room-temperature magnetic semiconductor and has great application potential in the field of spintronics in the future.

Further, we have studied the optoelectronic property of ultrathin (~15 nm) SnSe nanosheet (synthesized at 360 °C. The linear *I*_ds_-*V*_ds_ curve of SnSe device under 808 nm laser with different power densities indicated the perfect ohmic behavior (Fig. 6g). In particular, the device exhibited a maximum responsivity of 205 A/W (Fig. 6h) which is higher than SnSe nanomaterials obtained by magnetron sputtering technique and mechanical exfoliation at similar thickness^38,39^. The device demonstrated obvious switching behavior with a response time of 0.7 s and decay time of 0.3 s for the device (Fig. 6i, j) and the response speed is faster than the previous report, indicating the low defect level of our samples^40^. In addition, we also investigated the optoelectronic properties of MnTe nanosheets (synthesized at 440 °C) (Supplementary Fig. 43), which also showed an order of magnitude faster response speed than that of previous report^41^. Together, these studies clearly demonstrate that these low-temperature growth 2DMs exhibit high respectable electronic properties comparable to those prepared at much higher temperature. It could thus open up opportunities for BEOL integration with traditional semiconductor technology.

## Discussion

In this study, we have developed a BiOCl-assisted CVD method for the general synthesis of a diverse range of high-quality, ultrathin 2DMs from both LMs and NLMs at a growth temperature of about 280–500 °C. Significantly, the growth temperature is 200–300 °C lower than typical CVD process employed to grow 2DMs to date, and falls into the temperature range required by BEOL process in semiconductor processes. Our work shows the thickness and the size of high-quality 2DMs can be tuned by adjusting the growth time and duration. Detailed growth mechanism analysis reveals that BiOCl can decrease the volatilization temperature of the reactants, and the low-temperature process kinetically benefits 2D growth to achieve ultrathin 2DMs from both LM and NLMs. This oxygen-inhibited mechanism further promotes anisotropic growth NLMs to achieve 2D NLMs. The resulting 2DMs demonstrate excellent electrical, magnetic and optoelectronic property, further confirming the high crystal quality of the materials. The general low-temperature growth method could greatly enrich 2DMs family for exploring exotic physics and facile integration in existing semiconductor technology.

## Methods

### CVD growth

2D telluride, selenide, sulfide, oxide, and metal were all grown in a CVD system using ultrahigh purity Ar (purity 99.999%) and H_2_ (purity 99.999%) as the carrier gas and mica as the growth substrate (the growth diagram is shown in Supplementary Figs. S1 and S2). For 2D telluride, selenide, and sulfide, we used a dual temperature zone tube furnace as the reaction instrument. Te, Se, and S powders were placed upstream of the quartz tube, metal powder and basic salt BiOCl were ground and mixed thoroughly and placed downstream of the quartz tube. For 2D oxides and metals, we use a single-temperature zone tube furnace as a reactor to thoroughly grind and mix the metal powder and basic salt BiOCl and place them downstream of the quartz tube. Before the experiment, high flow Ar (500–800 sccm) was used to purge the air in the tube furnace. The detailed growth conditions are as follows:

### Growth of SnTe

We use a dual temperature zone tube furnace as an experimental instrument. Te powder (0.02 g, purity 99.99%) was placed in a quartz boat located in the center of the upstream furnace. Sn powder (0.18 g, purity 99.99%) and BiOCl (0.01 g, purity 99.99%, Alpha) powder were mixed uniformly and put into a quartz boat. The mica substrate was placed above the quartz boat and placed in the center of the heating area downstream of the quartz tube. During the growth process, a constant flow of 66 sccm Ar and 1 sccm H_2_ was used as the carrier gas. Then, the upstream and downstream regions were heated to 580 °C and 290~350 °C under ambient pressure, respectively, and after reaching the desired growth temperature, the temperature was maintained for 12 min. After the growth process was terminated, the furnace was cooled naturally to room temperature, and the sample was removed.

### Growth of In_2_Te_3_

The growth process of In_2_Te_3_ is similar to that of SnTe. The difference is that 0.05 g In powder (purity 99.9%) and 0.006 g BiOCl (purity 99.99%) powder are used; 45 sccm Ar and 1 sccm H_2_ are used; the temperature of the downstream area is 280~420 °C; and the growth time is 8 min.

### Growth of Cu_7_Te_4_

The growth process of Cu_7_Te_4_ is similar to that of SnTe. The difference is that 0.08 g Cu powder (purity 99.99%) and 0.01 g BiOCl (purity 99.99%) powder are used; 60 sccm Ar and 0.8 sccm H_2_ are used; the temperature of the downstream area is 400~480 °C; and the growth time is 12 min.

### Growth of CdTe

The growth process of CdTe is similar to that of SnTe. The difference is that 0.12 g Cd powder (purity 99.99%) and 0.01 g BiOCl (purity 99.99%) powder are used; 40 sccm Ar and 2 sccm H_2_ are used; the temperature of the downstream area is 440~540 °C; and the growth time is 20 min.

### Growth of MnTe

The growth process of MnTe is similar to that of SnTe. The difference is that 0.1 g Mn powder (purity 99.99%) and 0.02 g BiOCl (purity 99.99%) powder are used; 90 sccm Ar and 2 sccm H_2_ are used; the temperature of the downstream area is 440~520 °C; and the growth time is 18 min.

### Growth of FeTe

The growth process of FeTe is similar to that of SnTe. The difference is that 0.25 g Fe powder (purity 99.99%) and 0.02 g BiOCl (purity 99.99%) powder are used; 90 sccm Ar and 2 sccm H_2_ are used; the temperature of the downstream area is 450~590 °C, and the growth time is 10 min.

### Growth of MnSe

The growth process of MnSe is similar to that of SnTe. The difference is that 0.04 g Se powder (purity 98%), 0.2 g Mn powder (purity 99.99%) and 0.04 g BiOCl (purity 99.99%) powder are used; 130 sccm Ar and 2 sccm H_2_ are used; the temperatures of the upstream and downstream areas are 320 °C and 410~560 °C, respectively; and the growth time is 15 min.

### Growth of Cu_2_Se

The growth process of Cu_2_Se is similar to that of MnSe. The difference is that 0.1 g Se powder (purity 98%), 0.2 g Cu powder (purity 99.9%) and 0.02 g BiOCl (purity 99.99%) powder are used; 120 sccm Ar and 2 sccm H_2_ are used; the temperature of downstream area is 400~480 °C; and the growth time is 18 min.

### Growth of SnSe

The growth process of SnSe is similar to that of MnSe. The difference is that 0.1 g Se powder (purity 98%), 0.6 g Sn powder (purity 99.99%) and 0.02 g BiOCl (purity 99.99%, Alpha) powder are used; 130 sccm Ar and 1.5 sccm H_2_ are used; the temperature of downstream area is 300~360 °C; and the growth time is 12 min.

### Growth of SnSe_2_

The growth process of SnSe_2_ is similar to that of MnSe. The difference is that 0.3 g Se powder (purity 98%), 0.5 g Sn powder (purity 99.99%) and 0.01 g BiOCl (purity 99.99%) powder are used; 60 sccm Ar and 1.5 sccm H_2_ are used; the temperature of downstream area is 360~450 °C; and the growth time is 15 min.

### Growth of FeSe_2_

The growth process of FeSe_2_ is similar to that of MnSe. The difference was that 0.1 g Se powder (purity 99.99%), 0.6 g Fe powder (purity 99.99%) and 0.03 g BiOCl (purity 99.99%) powder were used; 80 sccm Ar and 3 sccm H_2_ were used; the temperature of the downstream area was 500~580 °C; and the growth time was 10 min.

### Growth of GeSe_2_

The growth process of GeSe_2_ is similar to that of MnSe. The difference was that 0.3 g Se powder (purity 98%), 0.5 g Ge powder (purity 99.99%) and 0.04 g BiOCl (purity 99.99%) powder were used; 55 sccm Ar and 2 sccm H_2_ were used; the temperature of the downstream area was 420~560 °C; and the growth time was 12 min.

### Growth of ZnSe

The growth process of ZnSe is similar to that of MnSe. The difference is that 0.1 g Se powder (purity 99.9%), 0.2 g Zn powder (purity 99.99%) and 0.01 g BiOCl (purity 99.99%) powder are used; 60 sccm Ar and 3 sccm H_2_ are used; the temperatures of the upstream and downstream areas are ~350 °C and 430~500 °C, respectively; and the growth time is 10 min.

### Growth of In_2_Se_3_

The growth process of In_2_Se_3_ is similar to that of MnSe. The difference is that 0.2 g Se powder (purity 99.9%), 0.2 g In powder (purity 99.99%) and 0.01 g BiOCl (purity 99.99%) powder are used; 55 sccm Ar and 3 sccm H_2_ are used; the temperature of the upstream and downstream area is ~350 °C and 380~530 °C, respectively; and the growth time is 15 min.

### Growth of *γ*-MnS

The growth process of *γ*-MnS is similar to that of SnTe. The difference is that 0.1 g S powder (purity 99.999%), 0.2 g Mn powder (purity 99.99%) and 0.04 g BiOCl (purity 99.99%) powder are used; 140 sccm Ar and 2 sccm H_2_ are used; the temperatures of the upstream and downstream areas are ~220 °C and 480~580 °C, respectively; and the growth time is 20 min.

### Growth of FeS_2_

The growth process of FeS_2_ is similar to that of *γ*-MnS. The difference is that 0.2 g S powder (purity 99.999%), 0.2 g Fe powder (purity 99.99%) and 0.01 g BiOCl (purity 99.99%) powder are used; 90 sccm Ar and 2 sccm H_2_ are used; the temperatures of the upstream and downstream areas are ~220 °C and 480~560 °C, respectively; and the growth time is 12 min.

### Growth of SnS_2_

The growth process of SnS_2_ is similar to that of *γ*-MnS. The difference is that 0.1 g S powder (purity 99.999%), 0.25 g Sn powder (purity 99.99%) and 0.02 g BiOCl (purity 99.99%) powder are used; 100 sccm Ar and 2 sccm H_2_ are used; the temperatures of the upstream and downstream areas are ~220 °C and 380~440 °C, respectively; and the growth time is 8 min.

### Growth of ZnS

The growth process of ZnS is similar to that of *γ*-MnS. The difference is that 0.2 g S powder (purity 99.999%, Alpha), 0.25 g Zn powder (purity 99.99%) and 0.03 g BiOCl (purity 99.99%) powder are used; 150 sccm Ar and 10 sccm H_2_ are used; the temperature of the upstream and downstream area is ~200 °C and 380~490 °C, respectively; and the growth time is 10 min.

### Growth of *β*-In_2_S_3_

The growth process of *β*-In_2_S_3_ is similar to that of *γ*-MnS. The difference is that 0.2 g S powder (purity 99.999%, Alpha), 0.25 g In powder (purity 99.99%) and 0.02 g BiOCl (purity 99.99%) powder are used; 60 sccm Ar and 5 sccm H_2_ are used; the temperature of the upstream and downstream area is ~200 °C and 400~560 °C, respectively; and the growth time is 10 min.

### Growth of MnO

We use a single temperature zone tube furnace as an experimental instrument. Mn powder (0.2 g, purity 99.99%) and BiOCl (0.09 g, purity 99.99%) powder were mixed uniformly and put into a quartz boat. The mica substrate was placed above the quartz boat and placed in the center of the heating area of the quartz tube. During the growth process, a constant flow of 200 sccm Ar and 5 sccm H_2_ was used as the carrier gas. Then, the furnace was heated to 500~590 °C under ambient pressure, and after reaching the desired growth temperature, the temperature was maintained for 6 min. After the growth process was terminated, the furnace was naturally cooled to room temperature, and the sample was removed.

### Growth of α-Fe_2_O_3_

The growth process of *α*-Fe_2_O_3_ is similar to that of MnO. The difference is that 0.3 g Fe powder (purity 99.99%) and 0.1 g BiOCl (purity 99.99%) powder are used; 100 sccm Ar and 3 sccm H_2_ are used; the temperature of the furnace is 420~550 °C; and the growth time is 12 min.

### Growth of α-Sb_2_O_3_

The growth process of *α*-Sb_2_O_3_ is similar to that of MnO. The difference is that 0.1 g Sb powder (purity 98%) and 0.06 g BiOCl (purity 99.99%) powder are used; 120 sccm Ar and 3 sccm H_2_ are used; the temperature of the furnace is 400~460 °C; and the growth time is 9 min.

### Growth of β-Sb_2_O_3_

The growth process of *β*-Sb_2_O_3_ is similar to that of MnO. The difference is that 0.15 g Sb powder (purity 98%) and 0.07 g BiOCl (purity 99.99%) powder are used; 90 sccm Ar and 3 sccm H_2_ are used; the temperature of the furnace is 400~470 °C; and the growth time is 7 min.

### Growth of In_2_O_3_

The growth process of In_2_O_3_ is similar to that of MnO. The difference is that 0.1 g In powder (purity 99.99%) and 0.05 g BiOCl (purity 99.99%) powder are used; 60 sccm Ar and 2 sccm H_2_ are used; the temperature of the furnace is 380~470 °C; and the growth time is 23 min.

### Growth of ZnO

The growth process of ZnO is similar to that of MnO. The difference is that 0.15 g Zn powder (purity 99.99%) and 0.05 g BiOCl (purity 99.99%) powder are used; 220 sccm Ar and 10 sccm H_2_ are used; the temperature of the furnace is 440~500 °C; and the growth time is 6 min.

### Growth of Cd

The growth process of Cd is similar to that of MnO. The difference is that 0.2 g Cd powder (purity 99.9%) and 0.05 g BiOCl (purity 99.99%) powder are used; 100 sccm Ar and 20 sccm H_2_ are used; the temperature of the furnace is 350~450 °C; and the growth time is 20 min.

### Intermediate product acquisition

After growing for 1–3 min in an oxygen-free environment, we quickly cooled the sample to room temperature to prevent the loss of intermediate.

### Thermogravimetric analysis

We used a thermogravimetric analyzer (TGA209F1, Netzsch) to perform thermogravimetric analysis of the samples. Each metal raw material was ground and mixed with the basic salt BiOCl separately and uniformly mixed. Then, 0.01 mg of the mixture was put into an alumina crucible and heated from room temperature to 700 °C at 10 K/min. Use ultrahigh purity Ar as the carrier gas with a flow rate of 10 ml/min.

### Characterization equipments

The synthesized 2D materials were characterized by optical microscopy (DP27, OLYMPUS), SEM-EDS (MIRA3 LMH, TESCAN), HRTEM (JEM-2100F, JEOL, 343, operating at 200 kV and equipped with an EDS system), XPS (ESCALAB 250Xi, charging referenced to the C 1 s peak at 284.8 eV), Raman spectroscopy (invia-reflex, Renishaw with 488 nm laser as the excitation source) and AFM (Bioscope system, BRUCKER). for characterization.

### Device fabrication and characterization

We used e-beam lithography to fabricate Hall bar devices with 50 nm Au for electrical contact on SiO_2_/Si substrate. The magnetoconductivity measurement was performed with the six-terminal Hall bar structure in a physical property measurement system (Quantum design) using a lock-in amplifier (Stanford SR830). We fabricated FET devices on SiO_2_/Si substrate using e-beam lithography with 50 nm Au electrode. The FET devices measurements were performed using an Agilent B1500A semiconductor analyser at room temperature. We fabricated photodetector devices directly on mica substrates using the transfer 50 nm Au electrode technique. The photodetector measurements were performed using an Agilent B1500A semiconductor analyser at room temperature. All measurements were performed in vacuum.

### Computational methodology

The Vienna ab initio simulation package (VASP)^42,43^ based on density functional theory (DFT) was used to simulate the formation energies for all structures. The projector augmented wave method^44,45^ and generalized gradient approximation^46^ with the Perdew-Burke-Ernzerhof realization were adopted for the core electrons. The cutoff energy and energy convergence criteria were set to 400 eV and 10^−4^ eV for all calculations. The experimental lattice parameters were used for all calculations, and the lateral dimensions of the supercell were set to larger than 12 Å. To avoid the interaction between adjacent surfaces, the thickness of vacuum space was approximately 15 Å. For all surface structures, we used a 6 × 6 × 1 Г-centered Monkhorst-Pack k-mesh for lattice relaxation. During the surface structure optimization, the inner three atom layers were fixed as the bulk phase, while the outermost three layers experienced atom relaxation for Cd and SnTe. For *γ*-MnS, the inner three Mn atom layers were fixed, while the outermost three Mn layers were relaxed. The surface formation energy was evaluated by the energy difference between the N layer for Cd (−67.916 eV), SnTe (−358.68 eV) and *γ*-MnS (−665.874 eV) unit cells and its counterpart with the N + 1 (adsorbed H/O/Cl atomic) layer^7^.

## Supplementary information

Supplementary Information

## Data Availability

Relevant data supporting the key findings of this study are available within the article and the Supplementary Information file. All raw data generated during the current study are available from the corresponding authors upon request.
