# Supplementary material for: General low-temperature growth of two-dimensional nanosheets from layered and nonlayered materials
Source: Nat Commun. 2023 Jan 19;14:304. doi: 10.1038/s41467-023-35983-6 (PMC9852450; doi:10.1038/s41467-023-35983-6)
Supplement: Supplementary file 1 — Supplementary Information [file 41467_2023_35983_MOESM1_ESM.pdf]

Supplementary Information

General low-temperature growth of two-dimensional nanosheets from layered and nonlayered materials

Biao Qin[1,‡], Muhammad Zeeshan Saeed[2,‡], Qiuqiu Li[2,‡], Manli Zhu[1,‡], Ya Feng[1,‡], Ziqi Zhou[3], Jingzhi Fang[3], Mongur Hossain[2], Zucheng Zhang[2], Yucheng Zhou[2], Ying Huangfu[2], Rong Song[2], Jingmei Tang[2], Bailing Li[2], Jialing Liu[2], Di Wang[2], Kun He[1], Hongmei Zhang[2], Ruixia Wu[2], Bei Zhao[2], Jia Li[2], Lei Liao[1], Zhongming Wei[3], Bo Li[1,4,*], Xiangfeng Duan[5], Xidong Duan[2,*]

[1]Hunan Provincial Key Laboratory of Two-Dimensional Materials, State Key Laboratory for Chemo/Biosensing and Chemometrics, Advanced Semiconductor Technology and Application Engineering Research Center of Ministry of Education of China, Changsha Semiconductor Technology and Application Innovation Research Institute, College of Semiconductors (College of Integrated Circuits), School of Physics and Electronics, Hunan University; Changsha 410082, China. [2]State Key Laboratory for Chemo/Biosensing and Chemometrics, College of Chemistry and Chemical Engineering, Hunan University; Changsha 410082, China. [3]State Key Laboratory of Superlattices and Microstructures, Institute of Semiconductors, Chinese Academy of Sciences, Beijing 100083, China. [4]Shenzhen Research Institute of Hunan University, Shenzhen 518063, China. [5]Department of Chemistry and Biochemistry, University of California, Los Angeles, CA, USA.

[‡]These authors contributed equally to this work.

* Email: boli@hnu.edu.cn; xidongduan@hnu.edu.cn

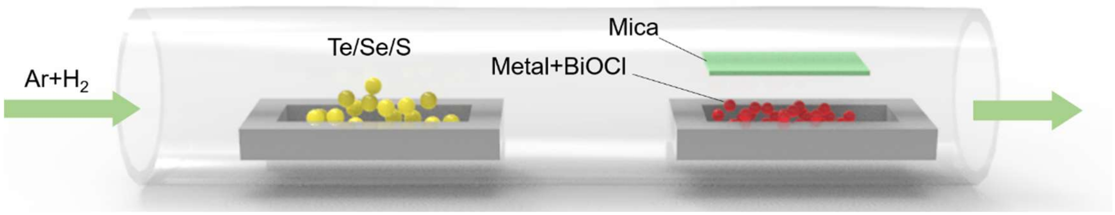

Supplementary Fig. 1 | Schematic diagram of the growth of 2D telluride, selenide and sulfide nanosheets in the BiOCl-assisted CVD system.

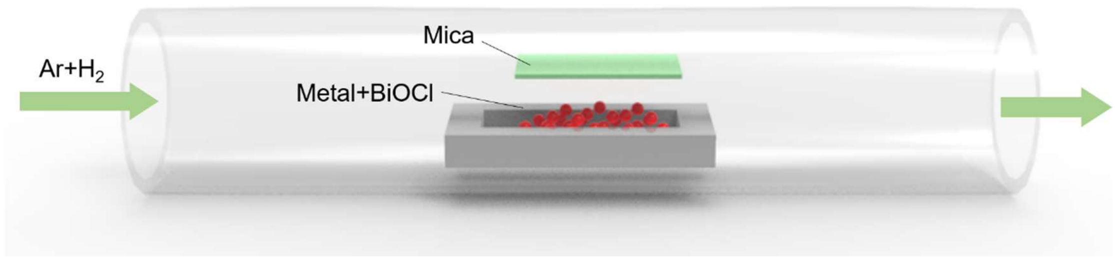

Supplementary Fig. 2 | Schematic diagram of the growth of 2D oxide and metal nanosheets in the BiOCl-assisted CVD system.

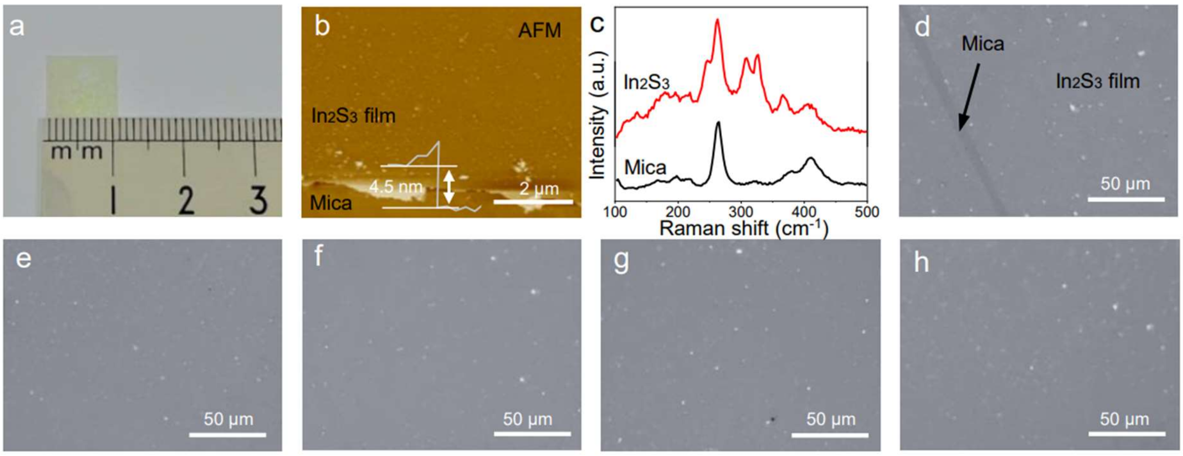

Supplementary Fig. 3 | The continuous uniform 2D In_2S_3 film. a, Photograph of 1×1 cm^2 few-layer In_2S_3 film on mica substrate. **b,** AFM image of the In_2S_3 film. **c,** Raman of the In_2S_3 film, red is In_2S_3 film, black is mica substrate. **d-h**, Optical microscope image of a continuous uniform film of In_2S_3 on a mica substrate.

Supplementary Table 1. Comparison of the synthesis temperature of the 2DMs grown by our method with other CVD methods without metal-organic precursors. ▲denotes this work.

**Metallic element**	**Materials**	**Synthesis temperature (℃)**	**Metallic element**	**Materials**	**Synthesis temperature (℃)**
Sn	SnTe	530,[1] 570,[2] 700,[3] 900 [4]	In	In_2Te_3	280~420▲
		290~350 ▲		In_2Te_3 ribbon	280~420▲
	SnSe	500,[5] 861 [6]		In_2O_3	380~470▲
		300~360 ▲		*β*-In_2S_3	700 [7]
	$SnSe_2$	550,[8] 600,[9] 642,[10] 750 [11]			400~560▲
		360~450 ▲		In_2Se_3	660,[12] 750,[13] 850 [14]
	SnS_2	650,[15] 700,[16] 710,[17] 850 [18]			380~530▲
		380~440 ▲	Ge	$GeSe_2$	420~560▲
Cd	Cd	350~450▲	Zn	ZnS	380~490 ▲
	CdTe	690 [19]		ZnSe	430~500 ▲
		440~540▲		ZnO	440~500▲
Mn	MnTe	580 [20]	Fe	$FeSe_2$	750 [21]
		440~520▲			500~580▲
	MnSe	590,[22] 680 [23]		FeTe	520,[24] 550,[25] 600 [26]
		410~560▲			450~590▲
	γ-MnS	480~580▲		FeS_2	640 [27]
	MnO	500~590▲			480~560▲
Sb	*α*-Sb_2O_3	650 [28]		*α*-Fe_2O_3	420~550▲
		400~460▲	Cu	Cu_7Te_4	400~480▲
	β-Sb_2O_3	650 [28]		Cu_2Se	400~480▲
		400~470▲			

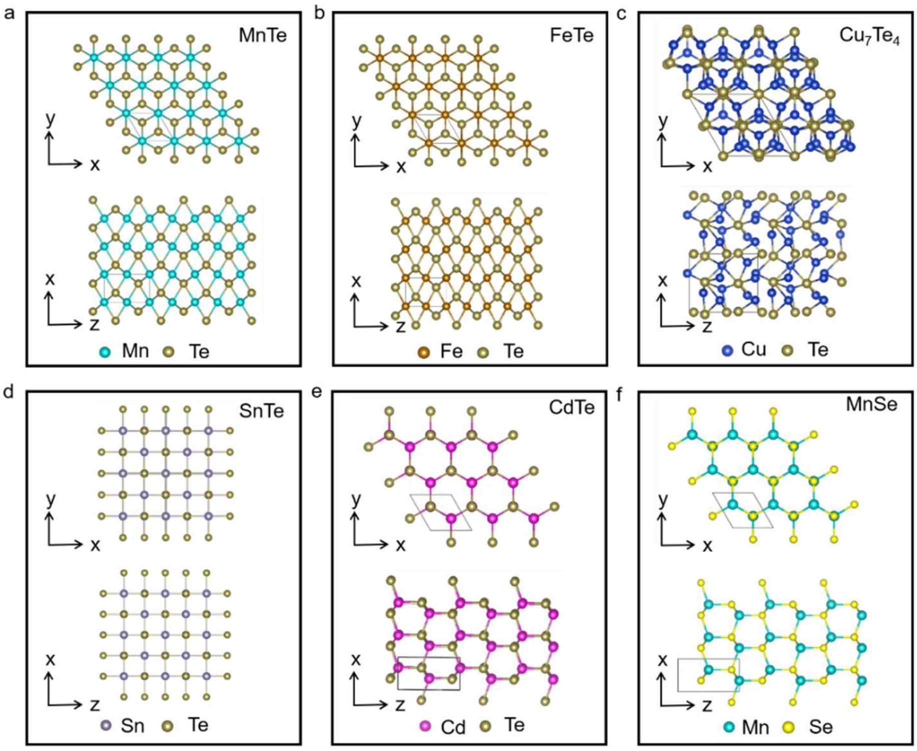

Supplementary Fig. 4 | Atomic structures of MnTe (a), FeTe (b), Cu_7Te_4 (c), SnTe (d), CdTe (e) and MnSe (f).

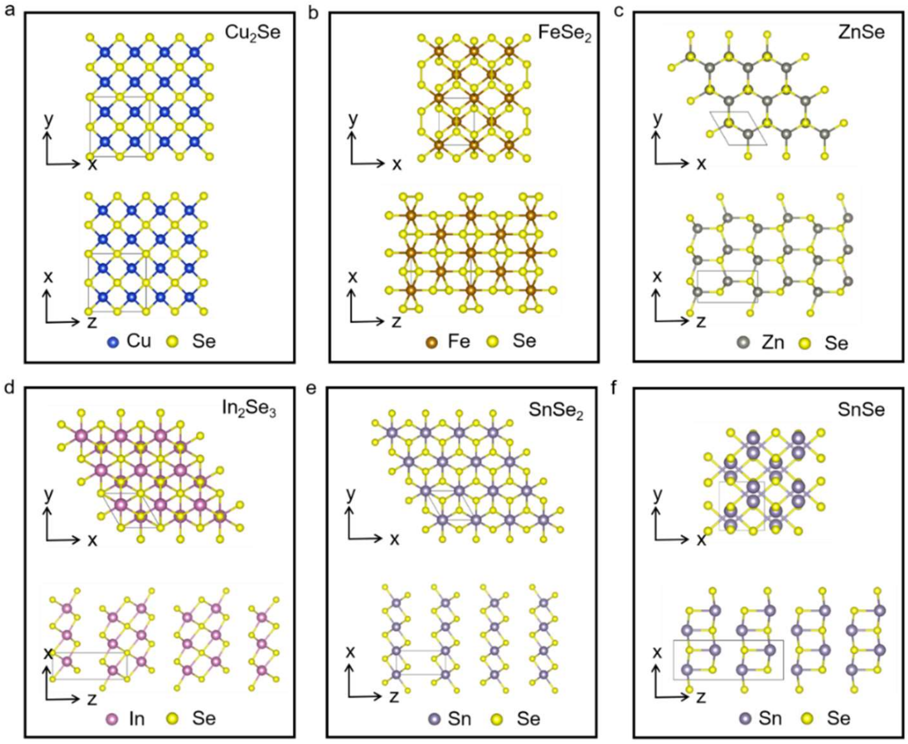

Supplementary Fig. 5 | Atomic structures of Cu_2Se (a), $FeSe_2$ (b), ZnSe (c), In_2Se_3 (d), $SnSe_2$ (e) and SnSe (f).

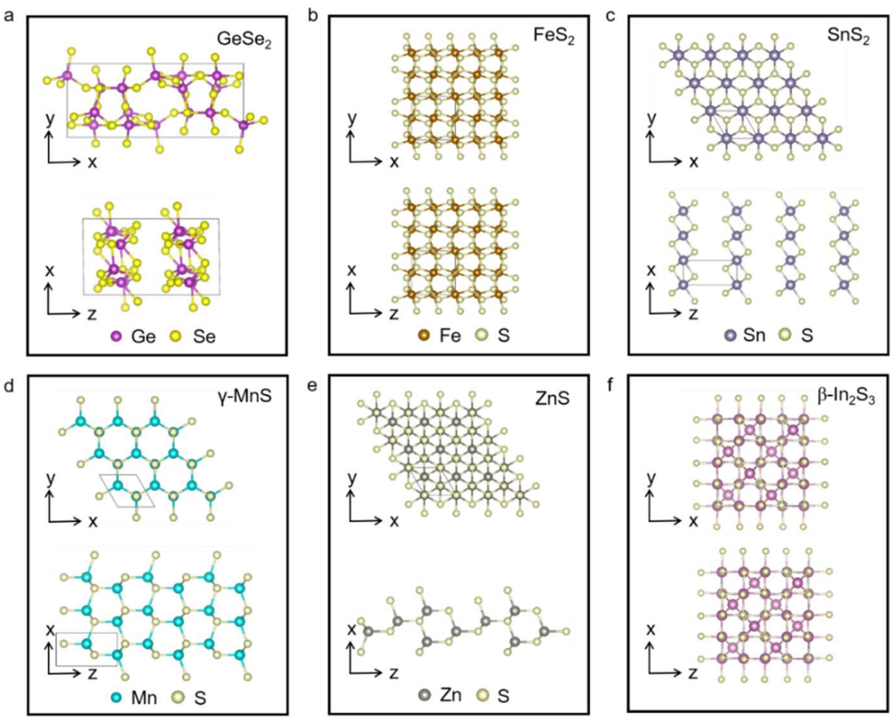

Supplementary Fig. 6 | Atomic structures of $GeSe_2$ (a), FeS_2 (b), SnS_2 (c), MnS (d), ZnS (e) and β-In_2S_3 (f).

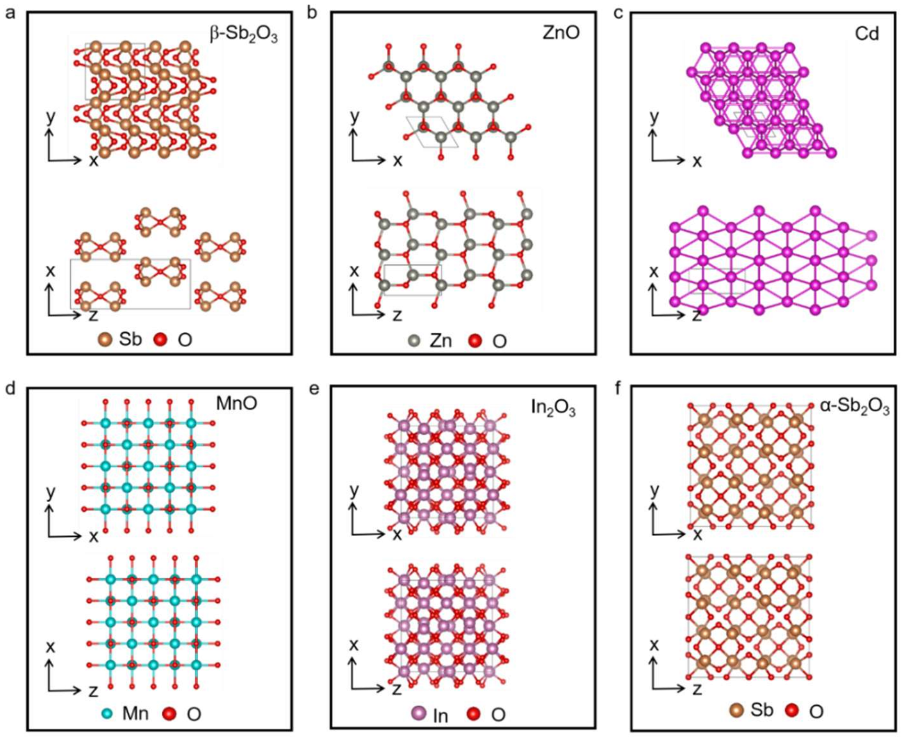

Supplementary Fig. 7 | Atomic structures of *β*-Sb_2O_3 (a), ZnO (b), Cd (c), MnO (d), In_2O_3 (e), and *α*-Sb_2O_3 (f).

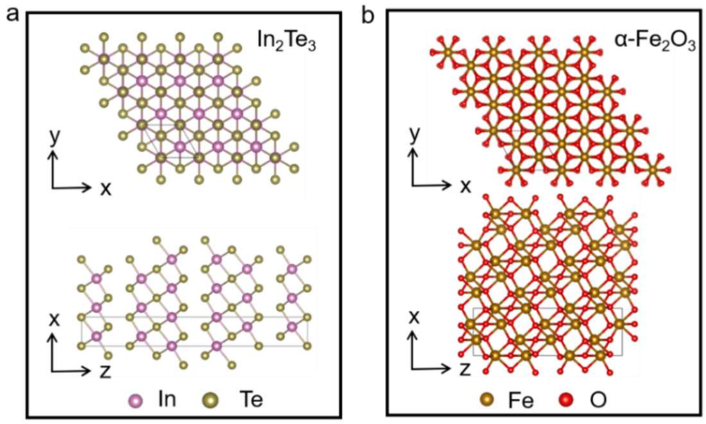

Supplementary Fig. 8 | Atomic structures of In_2Te_3 (a) and α-Fe_2O_3 (b).

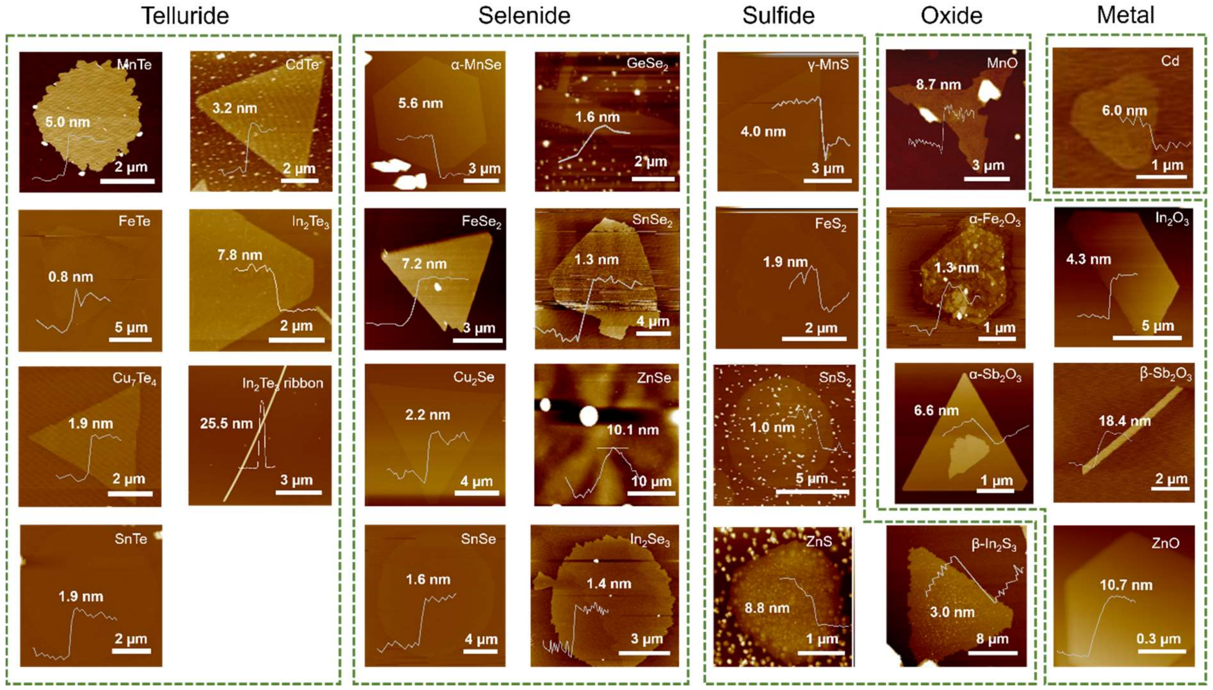

Supplementary Fig. 9 | Typical AFM images of the grown materials. The corresponding thickness data are shown by the white line.

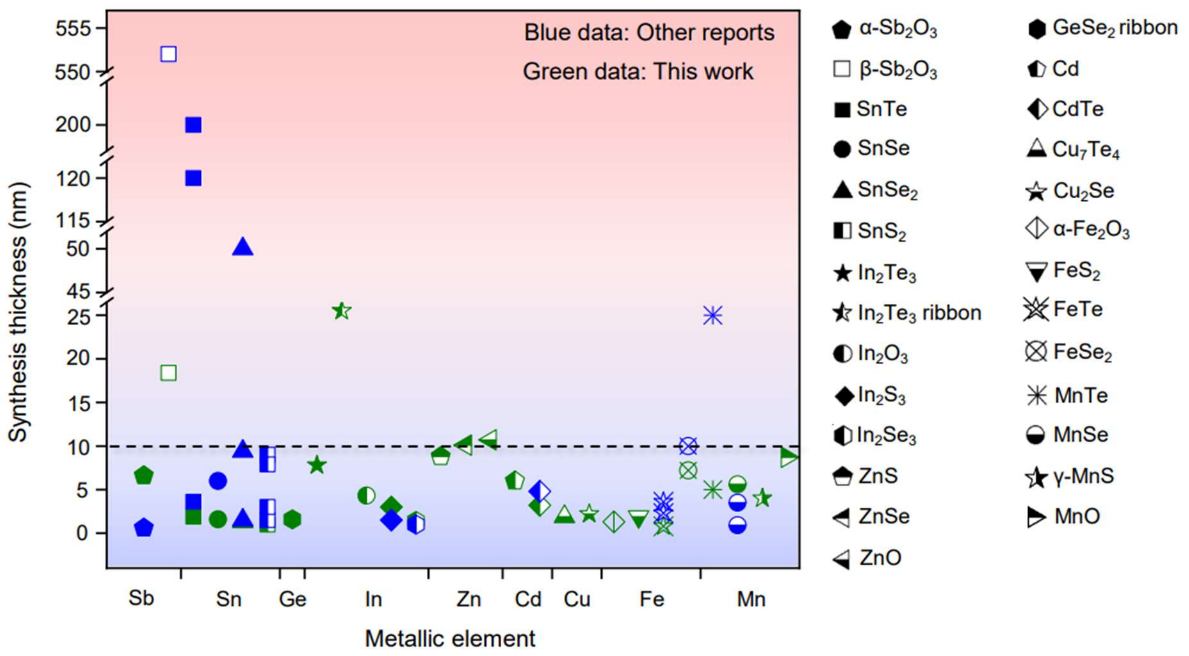

Supplementary Fig. 10 | Comparison of synthesis thickness of the material library with other reports. Most of the thinnest nanosheets were less than 10 nm. The blue data is other reports, green data is this work. Detailed references are shown in Supplementary Table 2.

Supplementary Table 2. Comparison of the synthesis thickness of the 2DMs grown by our method with other CVD methods. ▲denotes this work.

Metallic element	Materials	Structure	Synthesis, thickness (nm)	Metallic element	Materials	Structure	Synthesis, thickness (nm)
Sn	SnTe	NLM	3.6,[1] 120,[2] 200,[3]	In	In_2Te_3	LM	7.8▲
			1.9 ▲		In_2Te_3 ribbon	LM	25.5▲
	SnSe	LM	6,[5]		In_2O_3	NLM	4.3▲
			1.6 ▲		β-In_2S_3	NLM	1.5,[7]
	$SnSe_2$	LM	1.1,[9] 50,[8] 9.7,[11]				3▲
			1.3 ▲		In_2Se_3	LM	1,[12]
	SnS_2	LM	1.5,[15] 3,[17] 7.9,[18] 9,[16]				1.4▲

			1 ▲	Ge	$GeSe_2$	LM	1.6▲
Cd	Cd	NLM	6▲	Zn	ZnS	NLM	8.8 ▲
	CdTe	NLM	4.8,[19]		ZnSe	NLM	10.1 ▲
			3.2▲		ZnO	NLM	10.7▲
Mn	MnTe	NLM	25,[20]	Fe	$FeSe_2$	NLM	10,[21]
			5▲				7.2▲
	MnSe	NLM	0.9,[22] 3.5,[23]		FeTe	NLM	3.6,[24] 2.3,[25]
			5.6▲				0.8▲
	γ-MnS	NLM	4▲		FeS_2	NLM	1.9▲
	MnO	NLM	8.7▲				
Sb	α-Sb_2O_3	NLM	0.6,[28]				
					α-Fe_2O_3	NLM	1.3▲
			6.6▲	Cu	Cu_7Te_4	NLM	1.9▲
	β-Sb_2O_3	NLM	552,[28]		Cu_2Se	NLM	2.2▲
			18.4▲				

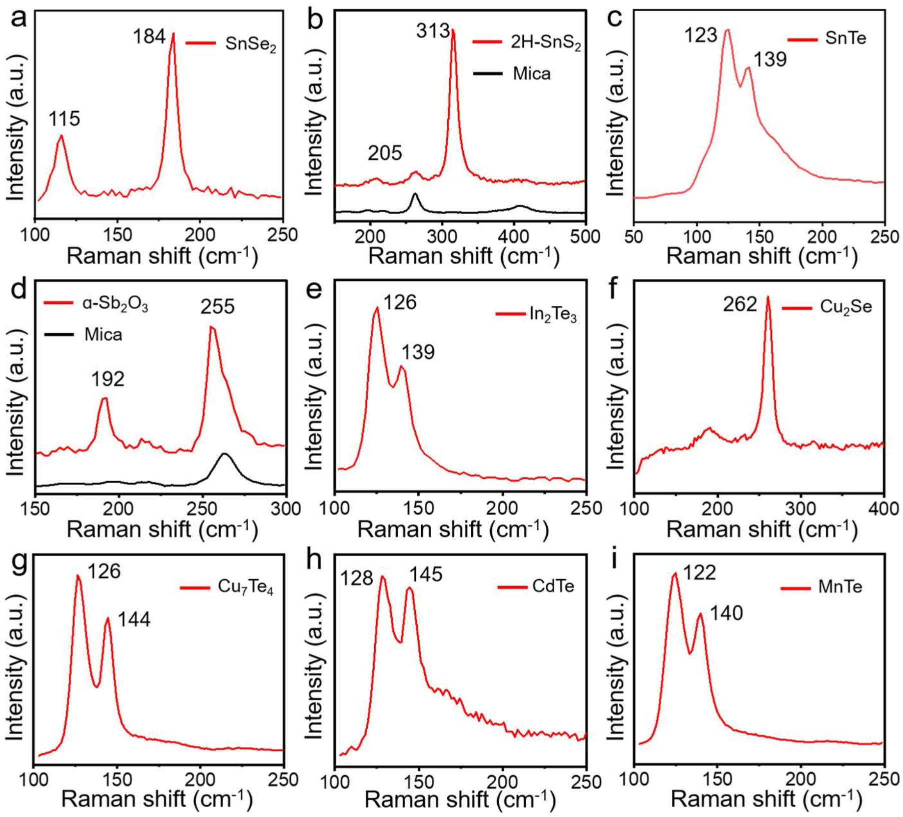

Supplementary Fig. 11 | Raman spectra of $SnSe_2$, 2H-SnS_2, SnTe, *α*-Sb_2O_3, In_2Te_3, Cu_2Se, Cu_7Te_4, CdTe, and MnTe nanosheets. Raman peaks of $SnSe_2$ are located at 115 and 184 cm^{-1}, corresponding to E_g and A_{1g} resonance modes[29]. The Raman spectra of 2H-SnS_2 are at 205 and 313 cm^{-1}, corresponding to E_g and A_{1g} resonance modes[15]. The Raman spectra of SnTe are at 123 and 139 cm^{-1}, corresponding to A_1 and E_{TO} resonance modes[1]. The Raman spectra of α-Sb_2O_3 are at 192 and 255 cm^{-1}, corresponding to F_{2g} and A_g resonance modes[30]. The Raman spectra of In_2Te_3 are at 126 and 139 cm^{-1}, corresponding to A_{1g} and E_g resonance modes[31]. Raman spectrum of the Cu_2Se are at 262 cm^{-1}[32]. The Raman spectra of Cu_7Te_4 are at 126 and 144 cm^{-1}[33]. The Raman spectra of CdTe are at 128 and 145 cm^{-1}, corresponding to A_1 and *LO* resonance modes[34]. The Raman spectra of MnTe are at 122 and 140 cm^{-1}, corresponding to A_{1g} and E_g resonance modes[35].

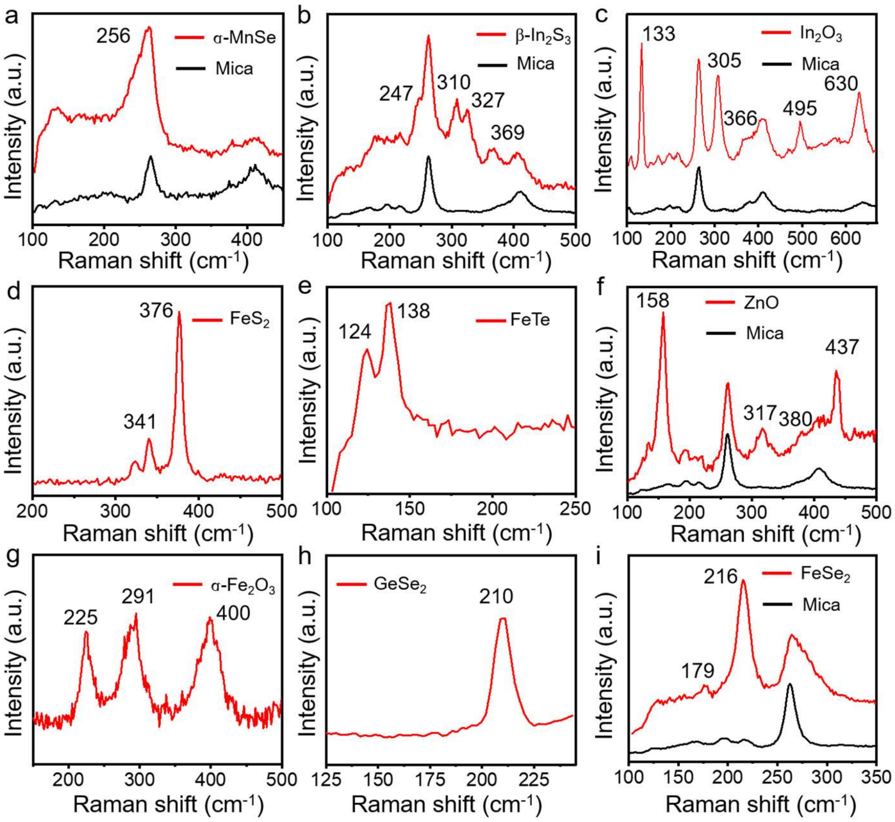

Supplementary Fig. 12 | Raman spectra of MnSe, *β*-In_2S_3, In_2O_3, FeS_2, FeTe, ZnO, *α*-Fe_2O_3, $GeSe_2$, and $FeSe_2$ nanosheets. The Raman spectrum of MnSe is at 256 cm^{-1}, corresponding to *LO* resonance modes[36]. The Raman spectra of *β*-In_2S_3 are at 247, 310, 327 and 369 cm^{-1}, corresponding to the A_{1g}, A_{1g}, F_{2g} and A_{1g} resonance modes[7]. The Raman spectra of In_2O_3 are at 133, 305, 366, 495 and 630 cm^{-1}, corresponding to E_{2g}, E_{1g}, E_{2g}, A_{1g} and E_{2g} resonance modes[37]. The Raman spectra of FeS_2 are at 341 and 376 cm^{-1}, corresponding to E_g and A_{1g} resonance modes[38]. The Raman spectra of FeTe are at 124 and 138 cm^{-1}, corresponding to E_g and A_{1g} resonance modes[39]. The Raman spectra of ZnO are at 158, 317, 380 and 437 cm^{-1}, corresponding to E_2, A_1^{TO} and E_g resonance modes[40]. The Raman spectra of *α*-Fe_2O_3 are at 225, 291 and 400 cm^{-1}, corresponding to the A_{1g}, E_g and E_g resonance modes[41]. The Raman spectrum of $GeSe_2$ is at 210 cm^{-1}, corresponding to A_g resonance modes[42]. The Raman spectra of $FeSe_2$ are at 179 and 216 cm^{-1}[43].

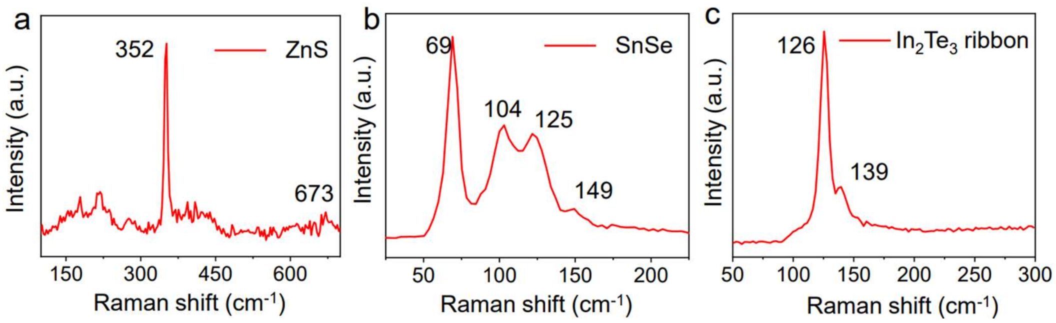

Supplementary Fig. 13 | Raman spectra of ZnS and SnSe nanosheets. The Raman spectrum of ZnS is at 352 cm^{-1} and 673 cm^{-1}, corresponding to *LO* resonance modes[44]. The Raman spectra of SnSe are at 69, 104, 125 and 149 cm^{-1}, corresponding to the A_{1g}, B_{3g}, A^2_g and A^3_g resonance modes[5]. The Raman spectra of In_2Te_3 are at 126 and 139 cm^{-1}, corresponding to A_{1g} and E_g resonance modes[31].

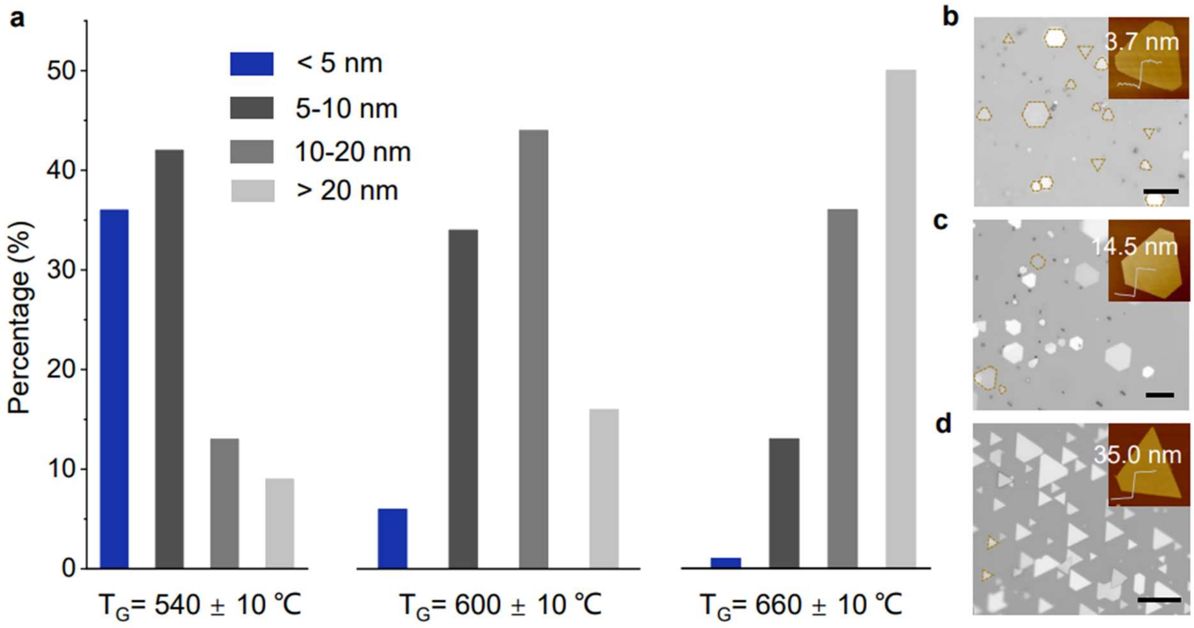

Supplementary Fig. 14 | Thickness-tunable synthesis of nanoplates by varying the growth temperature (T_G). a, The statistical thickness distributions of the α-Fe_2O_3 nanosheets synthesized with T_G set at 540 ± 10 °C, 600 ± 10 °C, and 660 ± 10 °C, respectively. **b-d,** Typical optical microscopy (OM) images and AFM image (inset) of the α-Fe_2O_3 nanosheets synthesized with T_G set at 540 °C(b), 610 °C(c), and 670 °C(d), respectively, under a constant carrier gas flow at 100 sccm (Ar/H_2 mixture with 3% H_2). Scale bar: 20 μm.

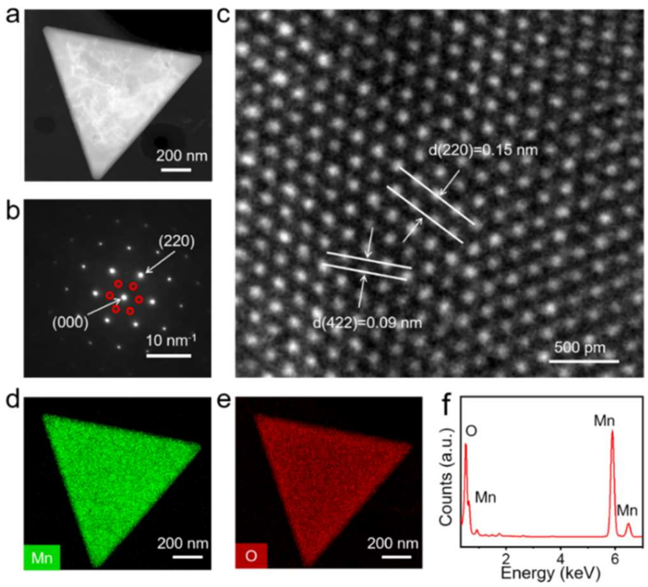

Supplementary Fig. 15 | TEM characterization of the atomic structure of MnO nanosheets. a, HAADF-TEM image of MnO nanosheet. **b,** The corresponding SAED image. **c,** The corresponding HRTEM image. It showed a hexagonal periodic arrangement, and the lattice spacings of the nanosheets were 0.15 and 0.09 nm, corresponding to the (220) and (422) planes of the hexagonal structure. **d, e,** TEM-EDS mapping of Mn and O in MnO nanosheets. **f,** The corresponding EDS spectrum of MnO nanosheets with a Mn:O atomic ratio of 1:1.

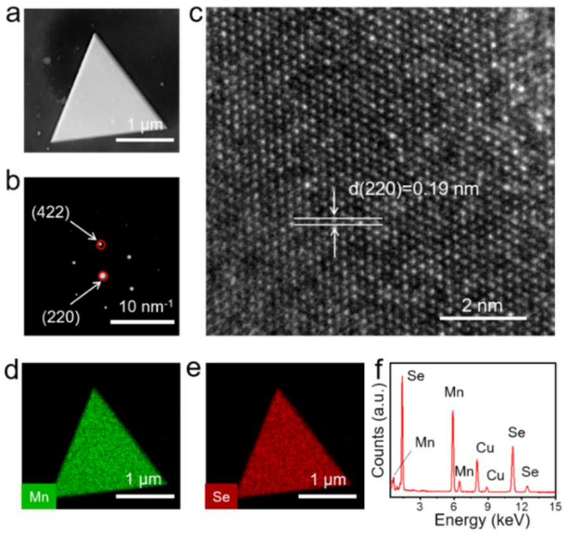

Supplementary Fig. 16 | TEM characterization of the atomic structure of MnSe nanosheets. a, HAADF-TEM image of MnSe nanosheet. **b,** The corresponding SAED image. **c,** The corresponding HRTEM image. The lattice spacing of the nanosheet is 0.19 nm, corresponding to the (220) planes of the hexagonal structure. **d, e,** TEM-EDS mapping of Mn and Se in MnSe nanosheets. **f,** The corresponding EDS spectrum of MnSe nanosheets with a

Mn:Se atomic ratio of 1:1.

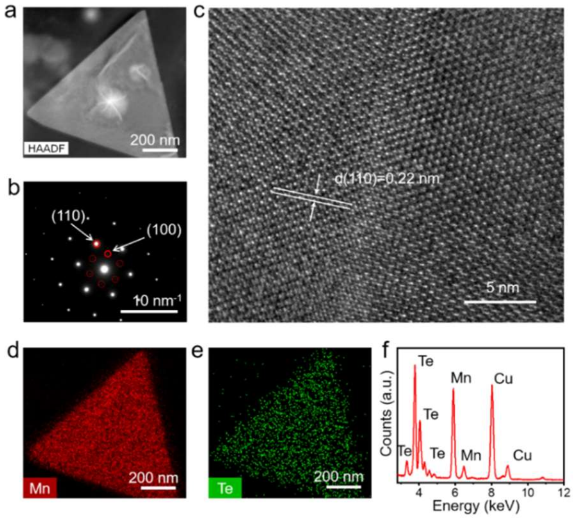

Supplementary Fig. 17 | TEM characterization of the atomic structure of MnTe nanosheets. a, HAADF-TEM image of MnTe nanosheet. **b,** The corresponding SAED image. **c,** The corresponding HRTEM image. It shows a hexagonal periodic arrangement, and the lattice spacing of the nanosheet is 0.22 nm, corresponding to the (110) planes of the hexagonal structure. **d, e,** TEM-EDS mapping of Mn and Te in MnTe nanosheets. **f,** The corresponding EDS spectrum of MnTe nanosheets with a Mn:Te atomic ratio of 1:1.

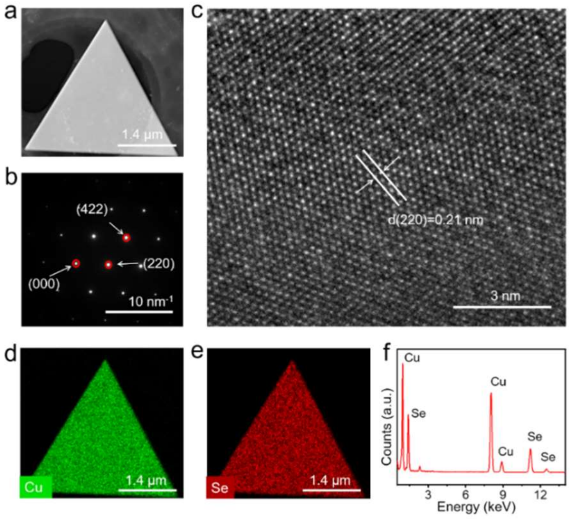

Supplementary Fig. 18 | TEM characterization of the atomic structure of Cu_2Se nanosheets. a, HAADF-TEM image of Cu_2Se nanosheet. **b,** The corresponding SAED image. **c,** The corresponding HRTEM image. It showed a hexagonal periodic arrangement, and the lattice spacing of the nanosheets was 0.21 nm, corresponding to the (220) planes of the

hexagonal structure. **d, e,** TEM-EDS mapping of Cu and Se in Cu_2Se nanosheets. **f,** The corresponding EDS spectrum of Cu_2Se nanosheets with a Cu:Se atomic ratio of 2:1.

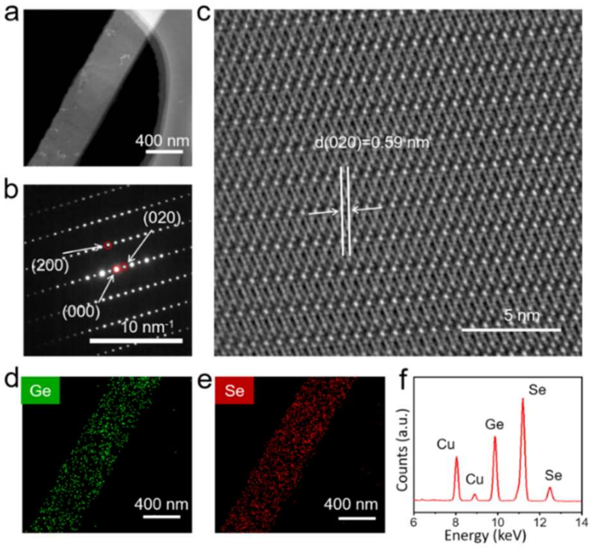

Supplementary Fig. 19 | TEM characterization of the atomic structure of $GeSe_2$ nanosheets. a, HAADF-TEM image of $GeSe_2$ nanosheet. **b,** The corresponding SAED image. **c,** The corresponding HRTEM image. It showed a tetragonal periodic arrangement, and the lattice spacing of the nanosheets was 0.59 nm, corresponding to the (020) planes of the tetragonal structure. **d, e,** TEM-EDS mapping of Ge and Se in $GeSe_2$ nanosheets. **f,** The corresponding EDS spectrum of $GeSe_2$ nanosheets on a copper mesh with carbon film. It shows that a Ge:Se atomic ratio of 1:2.

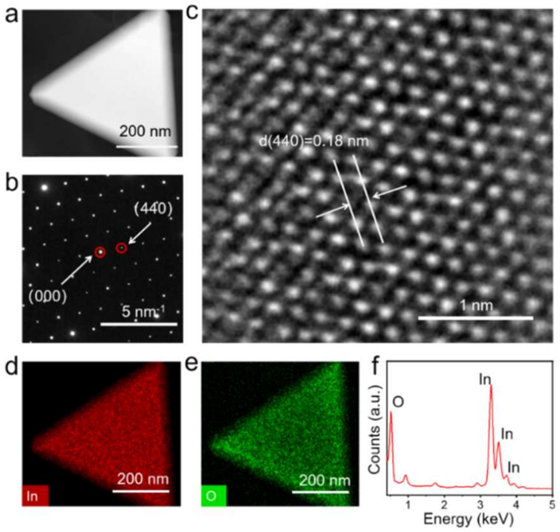

Supplementary Fig. 20 | TEM characterization of the atomic structure of In_2O_3

nanosheets. a, HAADF-TEM image of In_2O_3 nanosheet. **b,** The corresponding SAED image. **c,** The corresponding HRTEM image. It shows an almost perfect hexagonal periodic arrangement, and the lattice spacing of the nanosheet is 0.18 nm, corresponding to the (440) planes of the hexagonal structure. **d, e,** TEM-EDS mapping of In and O in In_2O_3 nanosheets. **f,** The corresponding EDS spectrum of In_2O_3 nanosheets with an In:O atomic ratio of 2:3.

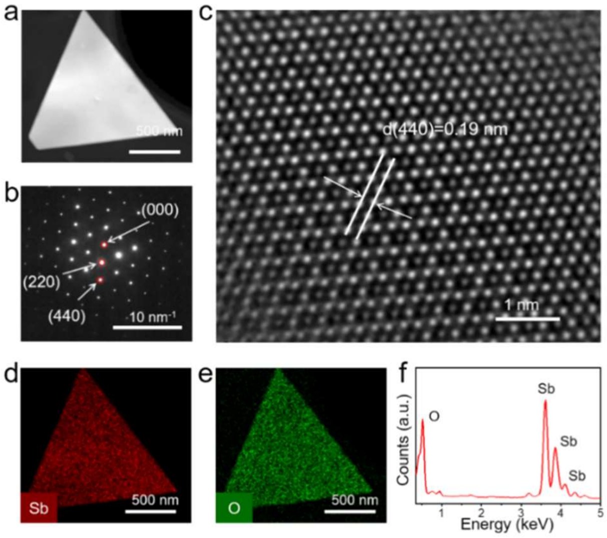

Supplementary Fig. 21 | TEM characterization of the atomic structure of α-Sb_2O_3 nanosheets. a, HAADF-TEM image of α-Sb_2O_3 nanosheet. **b,** The corresponding SAED image. **c,** The corresponding HRTEM image. It showed a hexagonal periodic arrangement, and the lattice spacing of the nanosheets was 0.19 nm, corresponding to the (440) planes of the hexagonal structure. **d, e,** TEM-EDS mapping of In and O in α-Sb_2O_3 nanosheets. **f,** The corresponding EDS spectrum of α-Sb_2O_3 nanosheets with an Sb:O atomic ratio of 2:3.

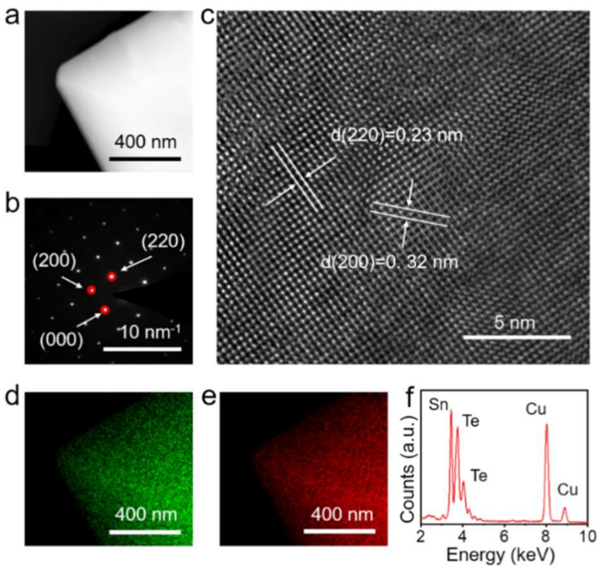

Supplementary Fig. 22 | TEM characterization of the atomic structure of SnTe nanosheets. a, HAADF-TEM image of SnTe nanosheet. **b,** The corresponding SAED image. **c,** The corresponding HRTEM image. It showed a hexagonal periodic arrangement, and the lattice spacings of the nanosheets were 0.23 and 0.32 nm, corresponding to the (220) and (200) planes of the hexagonal structure. **d, e,** TEM-EDS mapping of Sn and Te in SnTe nanosheets. **f,** The corresponding EDS spectrum of SnTe nanosheets with a Sn:Te atomic ratio of 1:1.

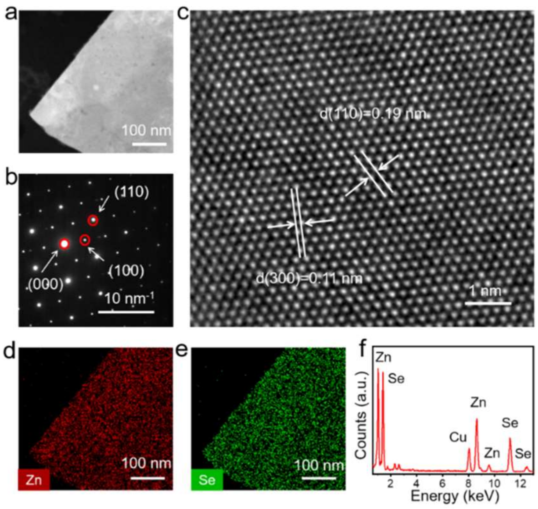

Supplementary Fig. 23 | TEM characterization of the atomic structure of ZnSe nanosheets. a, HAADF-TEM image of ZnSe nanosheet. **b,** The corresponding SAED image. **c,** The corresponding HRTEM image. It shows an almost perfect hexagonal periodic arrangement, and the lattice spacings of the nanosheets are 0.11 and 0.19 nm, corresponding to the (300) and (110) planes of the hexagonal structure. **d, e,** TEM-EDS mapping of Zn and Se in ZnSe nanosheets. **f,** The corresponding EDS spectrum of ZnSe nanosheets with a Zn:Se

atomic ratio of 1:1.

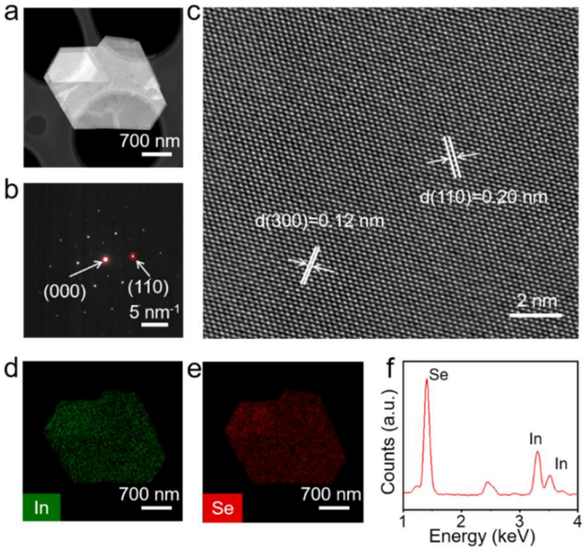

Supplementary Fig. 24 | TEM characterization of the atomic structure of In_2Se_3 nanosheets. a, HAADF-TEM image of In_2Se_3 nanosheet. **b,** The corresponding SAED image. **c,** The corresponding HRTEM image. It shows an almost perfect hexagonal periodic arrangement, and the lattice spacings of the nanosheets are 0.12 and 0.20 nm, corresponding to the (300) and (110) planes of the hexagonal structure. **d, e,** TEM-EDS mapping of In and Se in In_2Se_3 nanosheets. **f,** The corresponding EDS spectrum of In_2Se_3 nanosheets with an In:Se atomic ratio of 2:3.

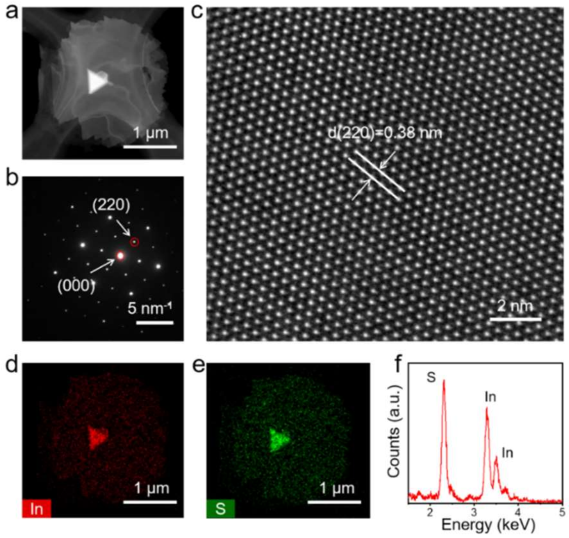

Supplementary Fig. 25 | TEM characterization of the atomic structure of β-In_2S_3 nanosheets. a, HAADF-TEM image of β-In_2S_3 nanosheet. **b,** The corresponding SAED

image. **c,** The corresponding HRTEM image. It showed an almost perfect hexagonal periodic arrangement, and the lattice spacing of the nanosheets was 0.38 nm, corresponding to the (220) planes of the hexagonal structure. **d, e,** TEM-EDS mapping of In and S in β-In_2S_3 nanosheets. **f,** The corresponding EDS spectrum of In_2Se_3 nanosheets with an In:S atomic ratio of 2:3.

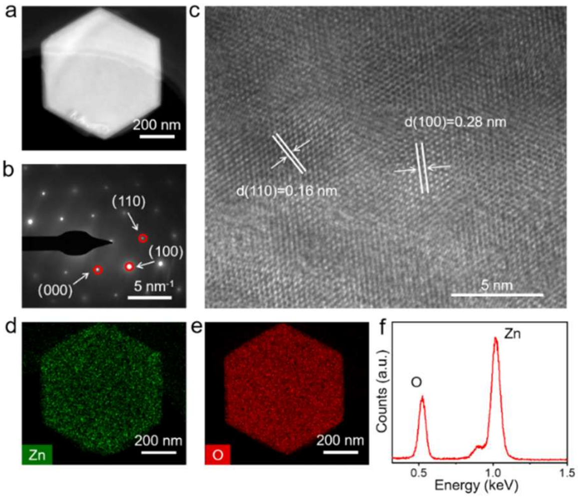

Supplementary Fig. 26 | TEM characterization of the atomic structure of ZnO nanosheets. a, HAADF-TEM image of ZnO nanosheet. **b,** The corresponding SAED image. **c,** The corresponding HRTEM image. It shows an almost perfect hexagonal periodic arrangement, and the lattice spacings of the nanosheets are 0.16 and 0.28 nm, corresponding to the (110) and (100) planes of the hexagonal structure. **d, e,** TEM-EDS mapping of Zn and O in ZnO nanosheets. **f,** The corresponding EDS spectrum of ZnO nanosheets with a Zn:O atomic ratio of 1:1.

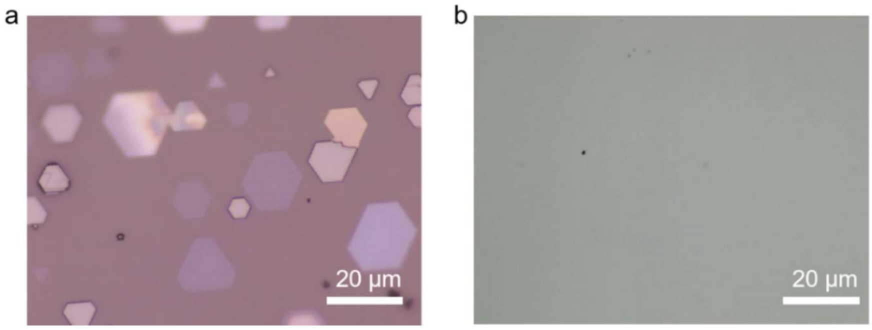

Supplementary Fig. 27 | Optical microscopy images of *α*-Fe_2O_3 grown with and without BiOCl (inset) under the same growth conditions on mica substrate.

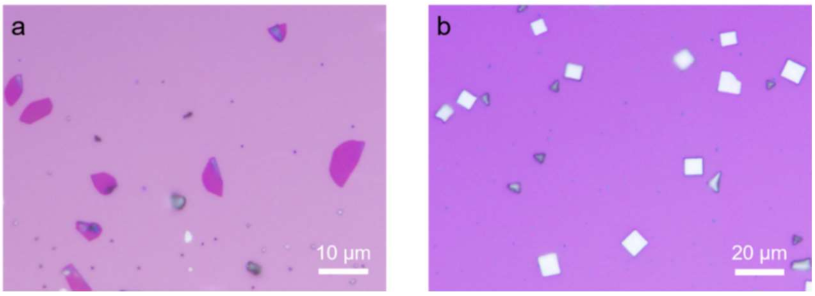

Supplementary Fig. 28 | (a) Optical microscopy images of SnTe grown on SiO_2/Si substrate with BiOCl. (b) Optical microscopy images of direct deposition of SnTe source material on SiO_2/Si substrate.

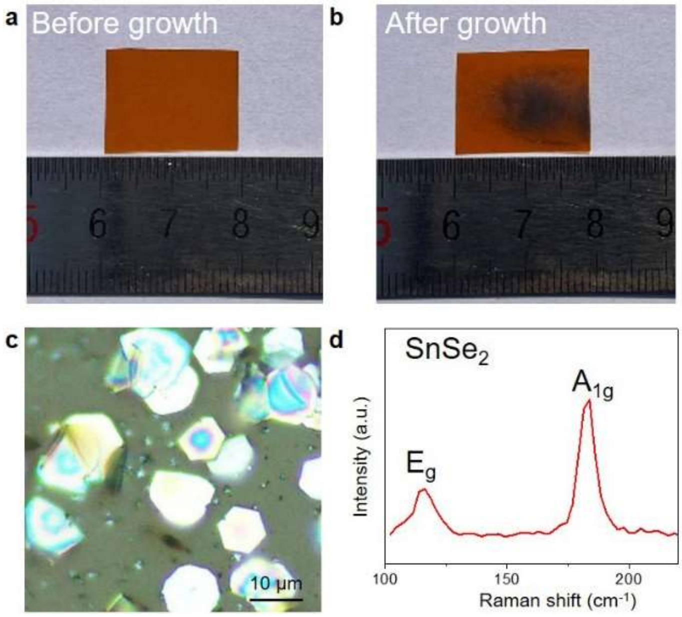

Supplementary Fig. 29 | The growth of $SnSe_2$ on PI substrate. Photos of PI substrate before growth (**a**) and after growth (**b**). **c**, Optical microscopy images of the $SnSe_2$ nanosheets on the PI substrate. **d**, Raman spectrum of the synthesized $SnSe_2$ nanosheets on the PI substrate. 2D $SnSe_2$ nanosheets with hexagonal morphology and high quality were synthesized on the polyimide (PI) substrate at 350 °C. PI is a kind of polymer with imide repeating unit, which has the advantages of wide applicable temperature (-200~400 °C), chemical corrosion resistance, high strength and so on. Today, it has been widely used in aviation, aerospace, microelectronics, nano, liquid crystal, separation membrane, laser and other fields.

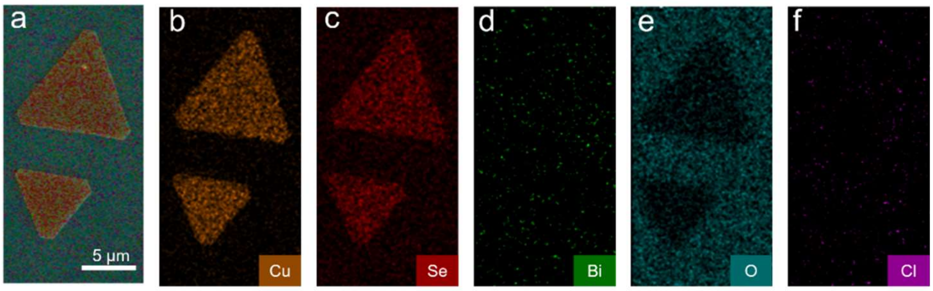

Supplementary Fig. 30 | SEM-EDS mapping images of synthesized 2D Cu_2Se nanosheets on mica substrate. EDS mapping showed that after the growth of Cu_2Se nanosheets, the nanosheets were composed of Cu and Se elements, and there were no Bi, O, or Cl elements in the nanosheets.

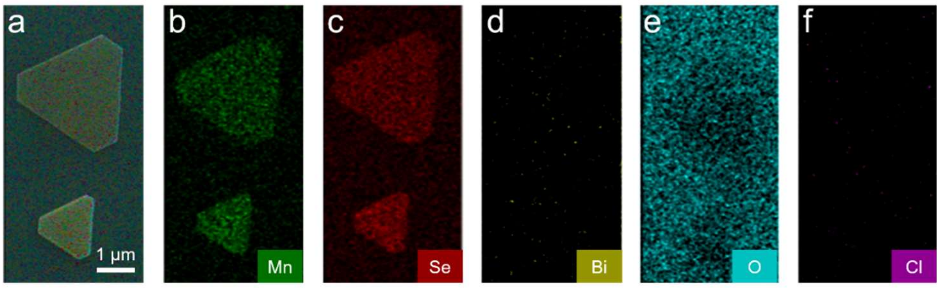

Supplementary Fig. 31 | SEM-EDS mapping image of synthesized 2D MnSe nanosheets on mica substrate. EDS mapping shows that after the growth of MnSe nanosheets, the nanosheets are composed of Mn and Se elements, and there are no Bi, O, or Cl elements in the nanosheets.

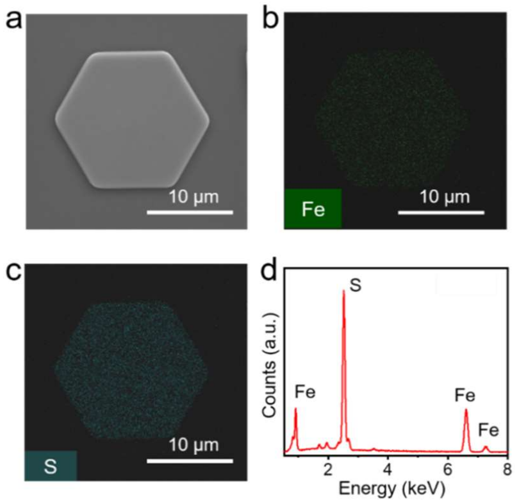

Supplementary Fig. 32 | SEM-EDS characterization of FeS_2 nanosheets on mica substrate. a, SEM image of the FeS nanosheets. **b, c,** SEM-EDS mapping of Fe and S in FeS

nanosheets. **d,** The corresponding EDS spectrum of FeS nanosheets with an Fe:S atomic ratio of 1:2.

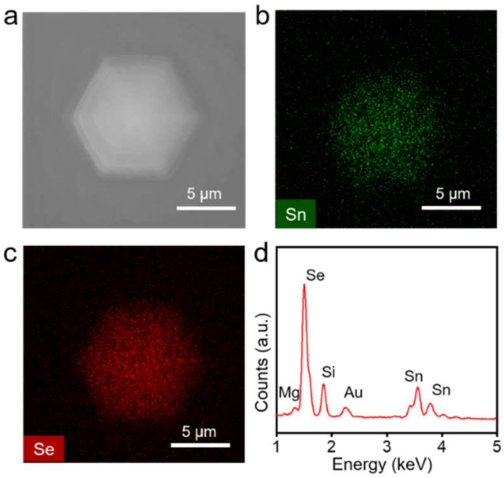

Supplementary Fig. 33 | SEM-EDS characterization of $SnSe_2$ nanosheets on mica substrate. a, SEM image of the $SnSe_2$ nanosheets. **b, c,** SEM-EDS mapping of Sn and Se in $SnSe_2$ nanosheets. **d,** The corresponding EDS spectrum of $SnSe_2$ nanosheets with a Sn:Se atomic ratio of 1:2.

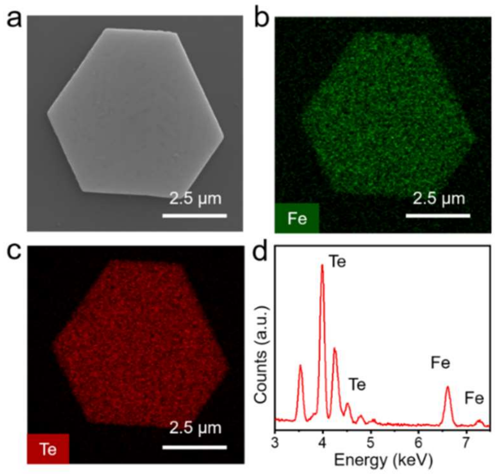

Supplementary Fig. 34 | SEM-EDS characterization of FeTe nanosheets on mica substrate. a, SEM image of the FeTe nanosheets. **b, c,** SEM-EDS mapping of Fe and Te in FeTe nanosheets. **d,** The corresponding EDS spectrum of FeTe nanosheets with a Sn:Se atomic ratio of 1:1.

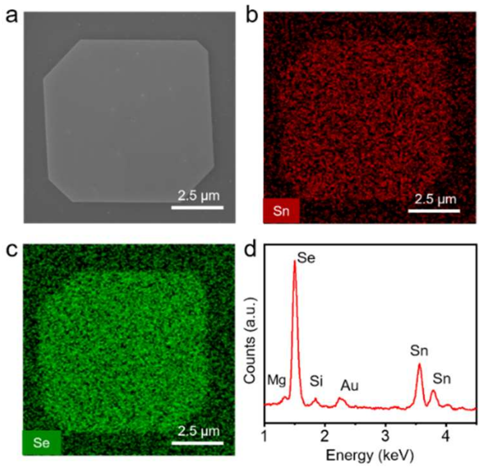

Supplementary Fig. 35 | SEM-EDS characterization of SnSe nanosheets on mica sbustrate. a, SEM image of the SnSe nanosheets. **b, c,** SEM-EDS mapping of Sn and Se in SnSe nanosheets. **d,** The corresponding EDS spectrum of SnSe nanosheets with a Sn:Se atomic ratio of 1:1.

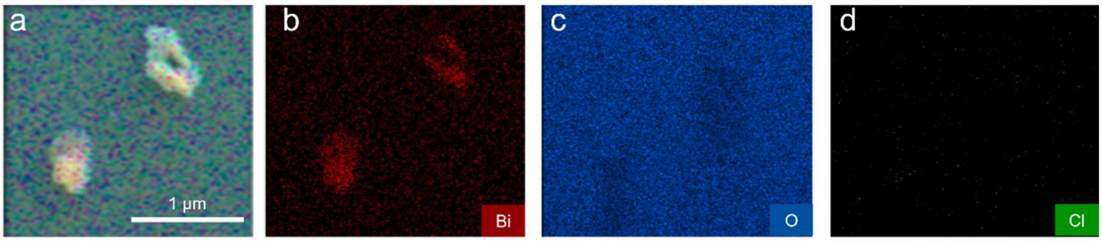

Supplementary Fig. 36 | SEM-EDS mapping image of the material obtained at the end of the reaction zone of the tube furnace (temperature-changing zone) after growing for 1 minute and rapidly cooling down on SiO_2/Si substrate.

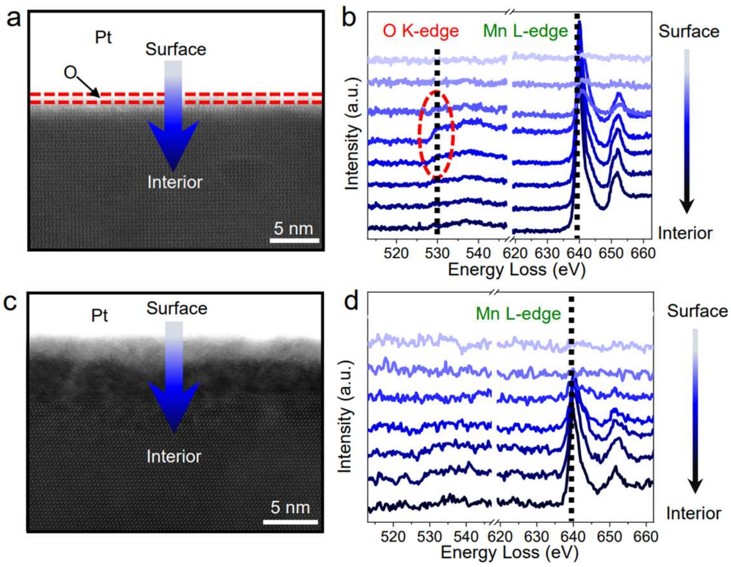

Supplementary Fig. 37 | Comparison the 2D MnS STEM and EELS of BiOCl-assisted growth method (a and b) and commonly used methods (c and d). For the BiOCl-assisted growth method, the clear atomic structure of MnS can be seen from STEM (**a**), and EELS (**b**) shows the content of O element from the outside to the interface, and then to the inside of the material. It can be seen that oxygen is present at the interface, and almost no oxygen is present at other positions. On the contrary, the MnS material obtained by the common method has ambiguous atomic structure (**c**) and surface interface, and EELS (**d**) shows that there is no oxygen element in the outside, the interface and the inside of the material.

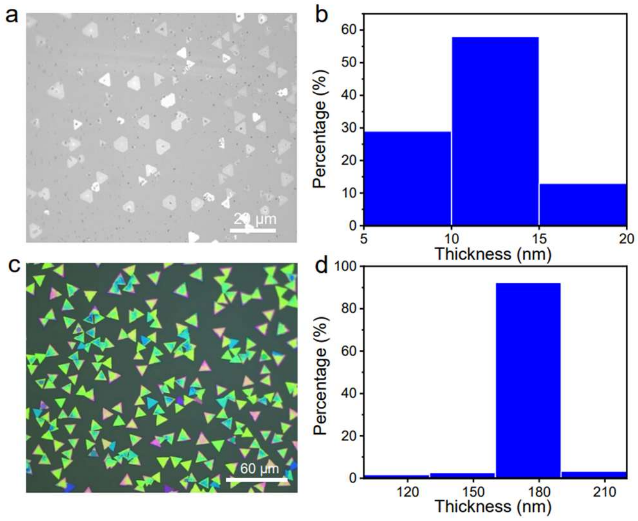

Supplementary Fig. 38 | Comparison 2D MnS thickness distribute of BiOCl-assisted growth method (a and b) and commonly used methods (c and d) under the same conditions. The thickness of nanosheets obtained under the same conditions by BiOCl-assisted growth method (**a** and **b**) method is less than 20 nm, while the thickness of nanosheets obtained by common method (**c** and **d**) is more than 100 nm or even 200 nm.

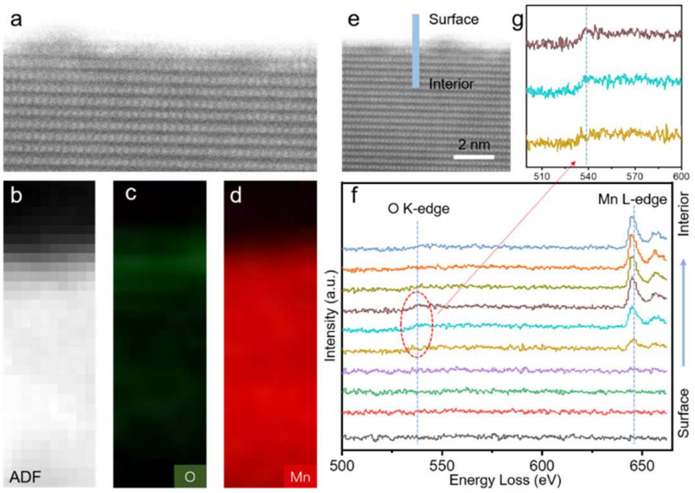

Supplementary Fig. 39 | Typical surface state and atomic structure of the sample. a, HAADF-STEM image of the cross-section of MnSe nanosheets. **b-d,** Annular dark field (ADF) image (**b**) and the corresponding distribution of O element (**c**) and Mn element (**d**) using MLLS fitting. **e,** HAADF-STEM image of the region from the surface to the interior in the cross-section of the MnSe nanosheet. **f,** Electron energy loss spectrum (EELS) spectrum corresponding to the O K-edge and Mn L-edge from the surface to the interior region. The blue arrow indicates the direction of the spectrum. **g,** EELS spectrum with enlarged area of O K-edge spectrum.

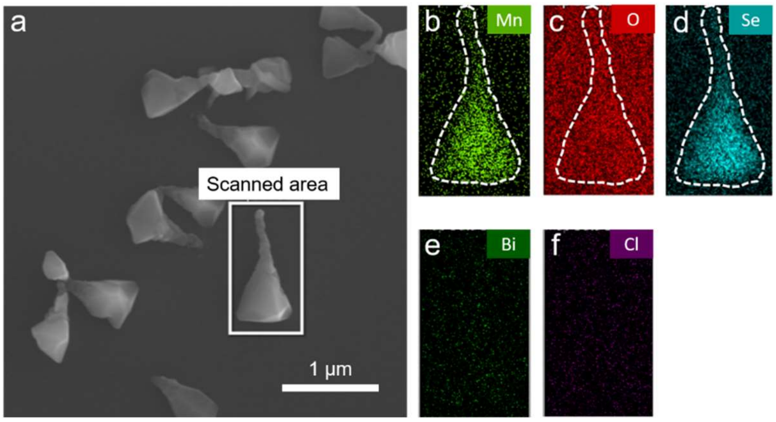

Supplementary Fig. 40 | SEM-EDS mapping image of the transition product obtained after growing in the reaction zone of the tube furnace for 1 minute and rapid cooling on mica substrate. SEM-EDS mapping showed that the transition product contains Mn, Se and O elements.

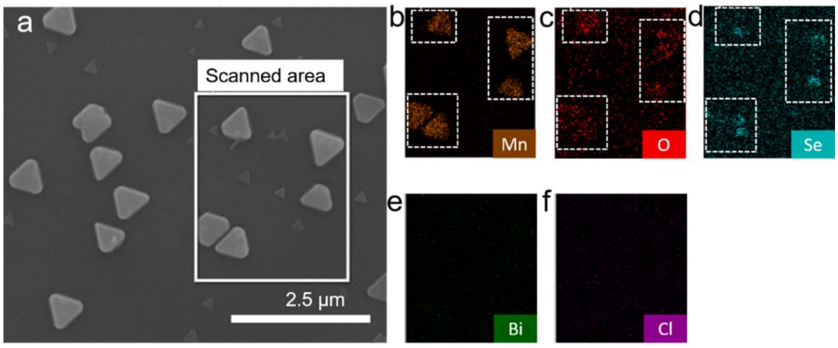

Supplementary Fig. 41 | SEM-EDS mapping image of the transition product obtained after growing in the reaction zone of the tube furnace for 2 minutes and rapid cooling on mica substrate. SEM-EDS mapping shows that the transition product contains Mn, Se and O elements.

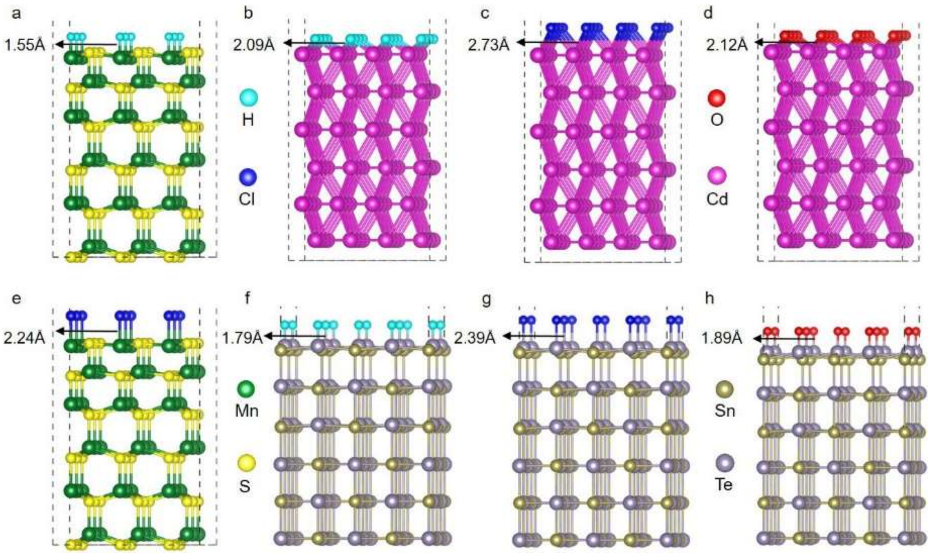

Supplementary Fig. 42 | Schematic view of adsorbing H, O and Cl atoms on the *γ*-MnS (001), Cd (001) and SnTe (001) surfaces. a, e, Schematic view of H and Cl atoms adsorbed on the surface of *γ*-MnS, respectively. **b-d,** Schematic view of H, Cl and O atoms adsorbed on the surface of Cd. **f-h,** Schematic view of H, Cl and O atoms adsorbed on the surface of SnTe.

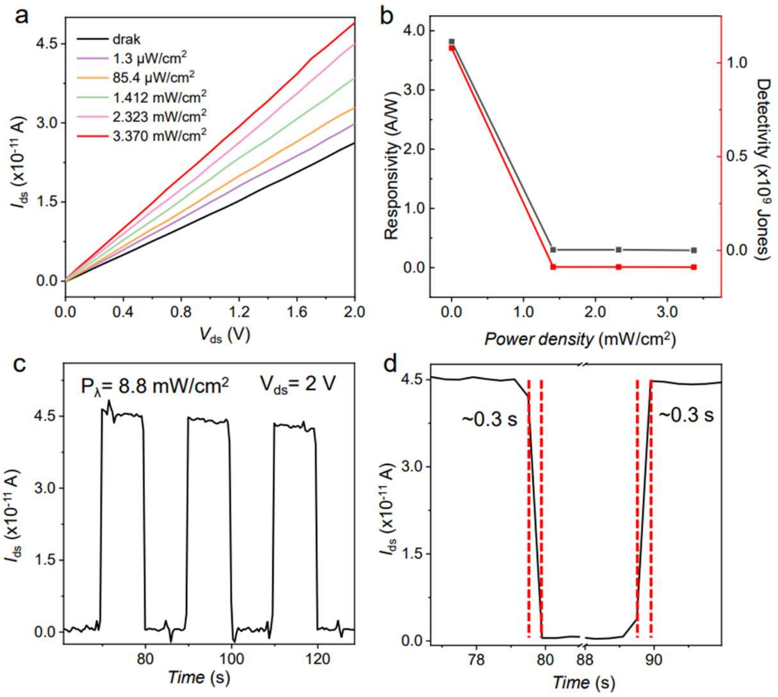

Supplementary Fig. 43 | Photoresponse of MnTe nanosheets photodetector at room temperature. a, I_{ds}–V_{ds} output characteristics of the MnTe photodetector in the dark and in the various laser powers with 450 nm laser. **b,** The photodetector responsivity (black line) and detectivity (red line) versus laser power densities with a voltage bias of 2 V. **c,** Time dependent

photoresponse of MnTe photodetector under 360 nm laser. Voltage bias is 2 V. **d,** A typical time-dependent photoresponse curve in a quick scan mode.

References

1 Liu, J. *et al.* Ultrathin high-quality SnTe nanoplates for fabricating flexible near-infrared photodetectors. *Acs Appl Mater Inter* **12**, 31810-31822 (2020).

2 Liu, P. Z. *et al.* Dislocation-driven SnTe surface defects during chemical vapor deposition growth. *J. Phys. Chem. Solids* **128**, 351-359 (2019).

3 Zou, Y. C. *et al.* Morphological control of SnTe nanostructures by tuning catalyst composition. *Nano Res.* **8**, 3011-3019 (2015).

4 Safdar, M. *et al.* Topological surface transport properties of single-crystalline SnTe nanowire. *Nano Lett.* **13**, 5344-5349 (2013).

5 Zhao, S. *et al.* Controlled synthesis of single-crystal SnSe nanoplates. *Nano Res.* **8**, 288-295 (2015).

6 Butt, F. K. *et al.* Synthesis of mid-infrared SnSe nanowires and their optoelectronic properties. *Crystengcomm* **16**, 3470-3473 (2014).

7 Huang, W. J. *et al.* Controlled synthesis of ultrathin 2D β-In_2S_3 with broadband photoresponse by Chemical Vapor Deposition. *Adv. Funct. Mater.* **27** (2017).

8 Wu, J. J. *et al.* Spiral growth of $SnSe_2$ crystals by chemical vapor deposition. *Adv. Mater. Interfaces* **3**, 1600383 (2016).

9 Zhang, Y. H. *et al.* Synthesis and surface-enhanced Raman scattering of ultrathin $SnSe_2$ nanoflakes by chemical vapor deposition. *Nanomaterials* **8** (2018).

10 Zhou, X. *et al.* Ultrathin $SnSe_2$ flakes grown by chemical vapor deposition for high-performance photodetectors. *Adv. Mater.* **27**, 8035-8041 (2015).

11 Huang, Y. *et al.* Designing the shape evolution of $SnSe_2$ nanosheets and their optoelectronic properties. *Nanoscale* **7**, 17375-17380 (2015).

12 Feng, W. *et al.* High-performance and flexible photodetectors based on chemical vapor deposition grown two-dimensional In_2Se_3 nanosheets. *Nanotechnology* **29**, 445205 (2018).

13 Rashid, R. *et al.* Shape-control growth of 2D-In_2Se_3 with out-of-plane ferroelectricity by chemical vapor deposition. *Nanoscale* **12**, 20189-20201 (2020).

14 Fu, X. *et al.* Controlled synthesis of carbon nanocoils on monolayered silica spheres. *Carbon* **99**, 43-48 (2016).

15 Xu, L. *et al.* Large-scale growth and field-effect transistors electrical engineering of atomic-layer SnS_2. *Small* **15**, e1904116 (2019).

16 Chen, Y. & Zhang, M. Large-area growth of SnS_2 nanosheets by chemical vapor deposition for high-performance photodetectors. *RSC Adv.* **11**, 29960-29964 (2021).

17 Su, G. X. *et al.* Chemical vapor deposition of thin crystals of layered semiconductor SnS_2 for fast photodetection application. *Nano Lett.* **15**, 506-513 (2015).

18 Wang, Z. G. & Pang, F. In-plane growth of large ultra-thin SnS_2 nanosheets by tellurium-assisted chemical vapor deposition. *RSC Adv.* **7**, 29080-29087 (2017).

19 Cheng, R. *et al.* Ultrathin single-crystalline CdTe nanosheets realized via Van der Waals epitaxy. *Adv. Mater.* **29**, 288-295 (2017).

20 Li, L. *et al.* Chemical vapor deposition-grown nonlayered *α*-MnTe nanosheet for photodetectors with ultrahigh responsivity and external quantum efficiency. *Chem. Mater.* **33**, 338-346 (2021).

21 Liu, H. T. & Xue, Y. Z. Van der waals epitaxial growth and phase transition of layered $FeSe_2$ nanocrystals. *Adv. Mater.* **33** (2021).

22 Zhang, Z. C. *et al.* Synthesis of ultrathin 2D nonlayered alpha-MnSe nanosheets, MnSe/WS_2 heterojunction for high-performance photodetectors. *Small Struct.* **2**, 2100028 (2021).

23 Zou, J. *et al.* Controlled growth of ultrathin ferromagnetic β-MnSe semiconductor. *SmartMat*, 1-9 (2022).

24 Kang, L. *et al.* Phase-controllable growth of ultrathin 2D magnetic FeTe crystals. *Nat. Commun.* **11**, 3729 (2020).

25 Cheng, M. *et al.* Phase-tunable synthesis and etching-free transfer of two-dimensional magnetic FeTe. *Acs Nano* **15**, 19089-19097 (2021).

26 Wang, X. G. *et al.* Ultrathin FeTe nanosheets with tetragonal and hexagonal phases synthesized by chemical vapor deposition. *Mater. Today* **45**, 35-43 (2021).

27 Zhou, J. D. *et al.* Composition and phase engineering of metal chalcogenides and phosphorous chalcogenides. *Nat Mater* (2022).

28 Han, W. *et al.* Two-dimensional inorganic molecular crystals. *Nat. Commun.* **10**, 4728 (2019).

29 An, B. X., Ma, Y., Zhang, G. Q., You, C. Y. & Zhang, Y. Z. Controlled synthesis of few-layer $SnSe_2$ by chemical vapor deposition. *Rsc Adv* **10**, 42157-42163 (2020).

30 Han, W. *et al.* Two-dimensional inorganic molecular crystals. *Nat. Commun.* **10**, 4728 (2019).

31 Zhang, S. *et al.* Large area growth of few-layer In_2Te_3 films by chemical vapor deposition and its magnetoresistance properties. *Sci. REP-UK* **9**, 10951 (2019).

32 MincevaSukarova, B., Najdoski, M., Grozdanov, I. & Chunnilall, C. J. Raman spectra of thin solid films of some metal sulfides. *J. Mol. Struct.* **410**, 267-270 (1997).

33 Salmon-Gamboa, J. U., Barajas-Aguilar, A. H., Ruiz-Ortega, L. I., Garay-Tapia, A. M. & Jimenez-Sandoval, S. J. Vibrational and electrical properties of $Cu_{2-x}Te$ films: experimental data and first principle calculations. *Sci. Rep-Uk* **8**, 8093 (2018).

34 Cheng, R. Q. *et al.* Ultrathin single-crystalline CdTe nanosheets realized via Van der Waals epitaxy. *Adv. Mater.* **29**, 1703122 (2017).

35 Li, L. J. *et al.* Chemical vapor deposition-grown nonlayered alpha-MnTe nanosheet for photodetectors with ultrahigh responsivity and external quantum efficiency. *Chem Mater* **33**, 338-346 (2021).

36 Popovic, Z. V. & Milutinovic, A. Far-infrared reflectivity and Raman scattering study of alpha-MnSe. *Phys Rev B* **73**, 155203 (2006).

37 Sans, J. A. *et al.* Structural and vibrational properties of corundum-type In_2O_3 nanocrystals under compression. *Nanotechnology* **28**, 205701 (2017).

38 Liang, Y. X. *et al.* An efficient precursor to synthesize various FeS_2 nanostructures via a simple hydrothermal synthesis method. *Crystengcomm* **18**, 6262-6271 (2016).

39 Kang, L. *et al.* Phase-controllable growth of ultrathin 2D magnetic FeTe crystals. *Nat. Commun.* **11**, 3729 (2020).

40 Silva, R. L. D. E. & Franco, A. Raman spectroscopy study of structural disorder degree of ZnO ceramics. *Mat. Sci. Semicon. Proc.* **119**, 105227 (2020).

41 Santillan, J. M. J. *et al.* Optical and magnetic properties of Fe nanoparticles fabricated by femtosecond laser ablation in organic and inorganic solvents. *Chemphyschem* **18**, 1192-1209 (2017).

42 Yan, Y. *et al.* Direct wide bandgap 2D $GeSe_2$ monolayer toward anisotropic UV photodetection. *Adv. Opt. Mater.* **7** (2019).

43 Shi, W. D., Zhang, X., Che, G. B., Fan, W. Q. & Liu, C. B. Controlled hydrothermal synthesis and magnetic properties of three-dimensional $FeSe_2$ rod clusters and microspheres. *Chem. Eng. J.* **215**, 508-516 (2013).

44 Fairbrother, A. *et al.* ZnS grain size effects on near-resonant Raman scattering: optical non-destructive grain size estimation. *Crystengcomm* **16**, 4120-4125 (2014).
